# Transitions between asynchronous and synchronous states: a theory of correlations in small neural circuits

**DOI:** 10.1007/s10827-017-0667-3

**Published:** 2017-11-10

**Authors:** Diego Fasoli, Anna Cattani, Stefano Panzeri

**Affiliations:** 1 Laboratory of Neural Computation, Center for Neuroscience and Cognitive Systems @UniTn, Istituto Italiano di Tecnologia, 38068 Rovereto, Italy; 2 Center for Brain and Cognition, Computational Neuroscience Group, Universitat Pompeu Fabra, 08002 Barcelona, Spain; 3 Department of Biomedical and Clinical Sciences “L. Sacco”, University of Milan, Milan, Italy

**Keywords:** Stochastic neural networks, Graded firing-rate model, Finite-size effects, Bifurcation analysis, Synchronous and asynchronous states, Propagation of chaos, Critical slowing down

## Abstract

The study of correlations in neural circuits of different size, from the small size of cortical microcolumns to the large-scale organization of distributed networks studied with functional imaging, is a topic of central importance to systems neuroscience. However, a theory that explains how the parameters of mesoscopic networks composed of a few tens of neurons affect the underlying correlation structure is still missing. Here we consider a theory that can be applied to networks of arbitrary size with multiple populations of homogeneous fully-connected neurons, and we focus its analysis to a case of two populations of small size. We combine the analysis of local bifurcations of the dynamics of these networks with the analytical calculation of their cross-correlations. We study the correlation structure in different regimes, showing that a variation of the external stimuli causes the network to switch from asynchronous states, characterized by weak correlation and low variability, to synchronous states characterized by strong correlations and wide temporal fluctuations. We show that asynchronous states are generated by strong stimuli, while synchronous states occur through critical slowing down when the stimulus moves the network close to a local bifurcation. In particular, strongly positive correlations occur at the saddle-node and Andronov-Hopf bifurcations of the network, while strongly negative correlations occur when the network undergoes a spontaneous symmetry-breaking at the branching-point bifurcations. These results show how the correlation structure of firing-rate network models is strongly modulated by the external stimuli, even keeping the anatomical connections fixed. These results also suggest an effective mechanism through which biological networks may dynamically modulate the encoding and integration of sensory information.

**Electronic supplementary material:**

The online version of this article (10.1007/s10827-017-0667-3) contains supplementary material, which is available to authorized users.

## Introduction

The study of correlations (or in general of statistical dependencies) among neurons is a topic of central importance to systems neuroscience. Statistical dependencies, which are also termed functional connectivity (Friston 2011), have been studied extensively at multiple scales, from interactions among neurons within a network, to interactions among macroscopic circuits (Sporns 2006; Bressloff 2009; Renart et al., 2010; Pernice et al., 2011; Trousdale et al., 2012; Buice & Chow 2013). There are several reasons why studying statistical interactions among neurons is important. Firstly, the structure of both second-order and higher-order statistical dependencies among neurons, and how these dependencies are modulated by either external stimuli or internal factors such as neuromodulation or attention, is key to understanding the information encoding capabilities of neural populations (Abbott & Dayan 1999; Pola et al., 2003; Pillow et al., 2008; Cohen & Maunsell 2009; Moreno-Bote et al., 2014). Secondly, measuring and understanding statistical dependencies is crucial to making inferences about how different neurons or areas exchange and integrate information (Singer 1993; Tononi et al., 1994; David et al., 2004; Rogers et al., 2007; Friston 2011). Thirdly, statistical dependencies among the activities of different neurons are useful to infer the underlying network structure (Friston et al., 2013; Gilson et al., 2016).

From the theoretical point of view, it is of particular interest to understand whether and how these statistical interactions can be modulated dynamically by changes in parameters, such as the strength of the external input to the network or by other network characteristics. This knowledge can help to better interpret patterns of correlations observed experimentally, and to understand how the brain can implement a dynamic qualitative change in information processing or transmission despite the relatively slow time scales of changes of anatomical connectivity (Womelsdorf et al., 2007; Akam & Kullmann 2010; Battaglia et al., 2012; Besserve et al., 2015). In their pioneering work (Ginzburg and Sompolinsky 1994), Ginzburg and Sompolinsky developed a first theory of correlations among neurons in a neural network model with binary firing rates. They proved that, in the limit of large network size, the population averaged activities can switch from asynchronous states (a regime in which correlations among neurons become weak and vanish as 1/*N*, where *N* is the size of the network) to synchronous states characterized by strong correlations.

The emergence of asynchronous states of uncorrelated neurons has been reported also by many other studies of large neural systems, and is key to the formulation of their mean-field approximation (Samuelides & Cessac 2007; Touboul et al., 2012; Baladron et al., 2012; Baladron Pezoa et al., 2012). On the other hand, synchronous regimes have been reported in large networks typically when the network undergoes critical slowing down, a phenomenon that happens when a system becomes increasingly sensitive to external perturbations (Kéfi et al., 2013). In this situation, the state variables undergo large and asymmetric fluctuations, with a strong increase in the cross- and auto-correlation functions (Scheffer et al., 2009; Kuehn 2013). Critical slowing down occurs at some (but not all) of the bifurcation points of the network’s dynamics, where small parameter variations cause profound qualitative changes in its dynamics. For example, in Ginzburg & Sompolinsky (1994) the authors showed the formation of critical slowing down in large networks of binary neurons when they approached a saddle-node or an Andronov-Hopf bifurcation, which corresponded to catastrophic transitions and the emergence of oscillatory activity, respectively.

The most established theories of correlation were developed in the large-network size limit (Ginzburg & Sompolinsky 1994; Bressloff 2009; Renart et al., 2010; Buice & Chow 2013) and can be in practice applied to macroscopic networks composed of at least few thousands of neurons. However, and somewhat counter-intuitively, the cross-correlation structure of small neural networks containing only a few tens of neurons can be much more difficult to study mathematically than that of large networks. This is mainly due to the impossibility to apply the powerful methods of statistical analysis, such as the law of large numbers and the central limit theorem, to small neural circuits. This fact prevents these theories to be able to describe networks of neurons at the mesoscopic and microscopic circuit level encompassing, for example, networks of few tens of cells in invertebrates (Williams & Herrup 1988). This small network level has been investigated in recent years both theoretically (Ingber^1992^; Freeman2000a, b; Wright *et al*. 2003; Bohland *et al*. 2009) and experimentally (Buzsáki et al., 2012; Einevoll et al., 2013), as it is a useful scale to link neural activity to brain function (Buzsáki & Draguhn 2004).

In this article we fill this gap by developing a theory of correlations in small neural circuits composed of homogeneous populations of fully-connected neurons. Unlike the analytical results introduced in Ginzburg & Sompolinsky (1994) and Renart et al. (2010), which were based on neurons with binary firing rates, we will consider the more complex and more biologically realistic case of graded firing rates. While Refs. (Ginzburg & Sompolinsky 1994; Renart et al., 2010) considered both fully-connected circuits with homogeneous synaptic weights and systems with random connectivity, for simplicity in this work we focus only on the case of networks with fully-connected topology (a possible extension to the case of random networks is discussed in Section 4.5). Moreover, unlike previous work, we will explicitly compute how cross-correlations relate to the whole bifurcation structure of the network. To do so, we study the dynamics of neural circuits of arbitrary size taking advantage of a mathematical approach that does not rely on statistical averaging (Fasoli et al., 2016). These networks of arbitrary size, when studied in the deterministic case, revealed a surprisingly rich set of local bifurcations of dynamics that could be studied analytically (Fasoli et al., 2016). By extending this previous work to include stochastic perturbations to the dynamics, we computed analytically the correlations among all neurons in the network, we studied their dependence on the network parameters and related them to the bifurcations of the dynamics.

We found that such finite-size networks displayed both asynchronous and synchronous regimes, with important qualitative and quantitative differences with respect to the large network size limit of Ref. (Ginzburg & Sompolinsky 1994). We proved that asynchronous states may occur also in small networks for strongly depolarizing or strongly hyperpolarizing stimuli, extending and generalizing previous analytical calculations (Fasoli et al., 2015) valid only when the network has a regular topology. We then proved the emergence in finite-size networks of critical slowing down at the saddle-node and Andronov-Hopf bifurcations, that was previously observed in the large-size limit of binary networks (Ginzburg & Sompolinsky 1994). Moreover, we proved that at the branching points or pitchfork bifurcations, which are characterized by a spontaneous symmetry- breaking of neural activity with spontaneous formation of heterogeneous activity within homogeneous populations, the inhibitory neurons undergo critical slowing down characterized by strong anti-correlation. This phenomenon was not found in large-size networks (Ginzburg & Sompolinsky 1994).

Interestingly, our formalism predicts the formation of synchronous and asynchronous states in networks composed of an arbitrary number of neural populations without calculating explicitly their cross-correlation structure (see the Online Resource 1 and Online Resource 2). However, our approach also allows explicit calculations in networks composed of a few neural populations. For exemplary purposes, in the main text we focus on the case of two neural populations, and we extensively validate the closed-form expression of the cross-correlations through numerical simulations.

## Materials and methods

### The stochastic firing-rate network model

Here we describe the stochastic firing-rate finite-size neural network model that we use in this article. This model is based on a number of assumptions and simplifications (described below) that represent the best compromise we could find between biological plausibility and mathematical tractability.

**Fig. 1:**
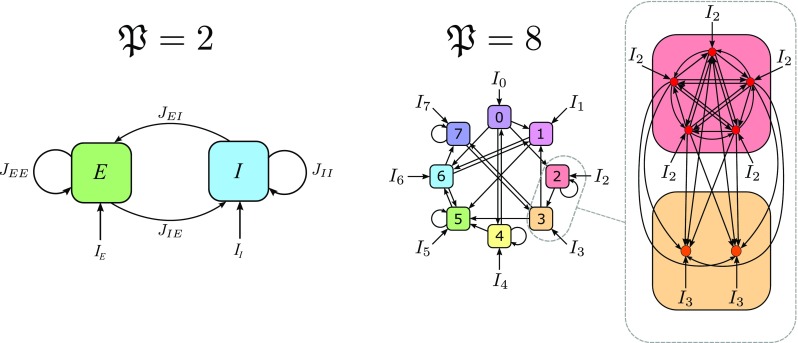
Examples of the neural network architectures considered here. The left panel shows the case of $\mathfrak {P}= 2$ populations that we study in the main text. For simplicity we identify the two populations by the letters *E*,*I*, in order to distinguish between populations of excitatory and inhibitory neurons. In the case $\mathfrak {P}= 2$ we consider non-zero synaptic weights, therefore the network has a fully-connected architecture (*M*_*E*_ = *M*_*I*_ = *N* − 1). The right panel shows an example of a network composed of $\mathfrak {P}= 8$ neural populations. In particular, we highlighted the structure of the synaptic connections within and between two neural populations. The extension of the theory to the case of an arbitrary number of neural populations has been developed in the Online Resource 1

A cortical column can be thought of as a network of neural masses distributed vertically across layers, and therefore it is composed of several populations of excitatory and inhibitory neurons (see for example (Binzegger et al., 2004)). Our theory can be used to study such cortical architectures, but the complexity of the resulting formulas increases considerably with the number of populations. Thus for exemplary purposes, in the main text we focus on the case of a network made of two fully homogeneous neural populations, one excitatory (*E*) and one inhibitory (*I*), which is commonly considered a good approximation of a single neural mass (Grimbert 2008). The structure of this network is schematized in the left-hand panel of Fig. 1. In the Online Resource 1 we generalize these results to an arbitrary number $\mathfrak {P}$ of populations (an example of network structure for $\mathfrak {P}= 8$ is shown in Fig. 1, right panel).

The populations contain an arbitrary finite number of neurons which are interconnected through synaptic connections with arbitrarily strong weights. In order to make the network analytically tractable, we assume (as it is often made when considering local cortical circuits Grimbert 2008; Deco et al., 2008) that the axonal delays are negligible. Moreover, whenever a neural population *α* projects synapses to a population *β* (with *α*,*β* = *E*,*I*), we assume that each neuron in population *α* sends connections to each neuron in population *β* (avoiding self-connections in the case *α* = *β*). Furthermore, as often done in theoretical neuronal network studies (Brunel & Hakim 1999; Steyn-Ross et al., 2004; Touboul et al., 2012), we describe random fluctuations in the network by means of a white noise component in the external input to the network.

In more detail, we consider a graded rate model to describe the dynamics of single neurons by means of the following system of stochastic differential equations:

1 $$ \begin{array}{ll} \frac{dV_{i}\left( t\right)}{dt}=&-\frac{1}{\tau_{i}}V_{i}\left( t\right)+\frac{1}{M_{i}}\sum\limits_{j = 0}^{N-1}J_{ij}\mathscr{A}_{j}\left( V_{j}\left( t\right)\right)+I_{i}\left( t\right)\\ &+\sigma_{i}^{\mathscr{B}}\frac{d\mathscr{B}_{i}\left( t\right)}{dt},\quad i = 0,...,N-1, \end{array} $$

where *N* is the number of neurons in the network, *V*_*i*_(*t*) is the membrane potential of the *i* th neuron at the time instant *t*, and *τ*_*i*_ is its membrane time constant. The normalization factor *M*_*i*_ represents the number of incoming connections to the *i* th neuron, while *J*_*ij*_ is the weight of the synaptic connection from the *j* th (presynaptic) neuron to the *i* th (postsynaptic) neuron. $\mathscr {A}_{j}\left (\cdot \right )$ is an algebraic activation function which converts the membrane potential *V* into the corresponding firing rate $\nu =\mathscr {A}\left (V\right )$, according to the formula:

2 $$ \mathscr{A}_{j}\left( V\right)=\frac{\nu_{j}^{\max}}{2}\left[1+\frac{\frac{\Lambda_{j}}{2}\left( V-{V_{j}^{T}}\right)}{\sqrt{1+\frac{{\Lambda_{j}^{2}}}{4}\left( V-{V_{j}^{T}}\right)^{2}}}\right]. $$

Here $\nu _{j}^{\max }$ is the maximum firing rate of the neuron, ${V_{j}^{T}}$ is the threshold of the activation function, and Λ_*j*_ is its slope parameter. The latter represents the “speed” with which the neuron switches between low rates (*ν*_*j*_ ≈ 0) and high rates ($\nu _{j}\approx \nu _{j}^{\max }$). Moreover, in Eq. (1) *I*_*i*_(*t*) is a deterministic external input (i.e. the stimulus) to the *i* th neuron, while $\sigma _{i}^{\mathscr {B}}\frac {d\mathscr {B}_{i}\left (t\right )}{dt}$ is a white noise input with normal distribution and standard deviation $\sigma _{i}^{\mathscr {B}}$. In order to apply linear response theory, we will assume that $\sigma ^{\mathscr {B}}$ is small enough to neglect second-order corrections to the perturbative expansion of the cross-correlations. This assumption will hold if the standard deviation of the fluctuations in the membrane potential is smaller than the minimum radius of curvature of the activation function of Eq. (2). The latter is a function of the parameters of the activation function: $r\left (\mu \right )=\left |\frac {\left (1+\mathscr {A}^{\prime }\left (\mu \right )^{2}\right )^{3/2}}{\mathscr {A}^{\prime \prime }\left (\mu \right )}\right |$, where $\mathscr {A}^{\prime }$ and $\mathscr {A}^{\prime \prime }$ are the first and second-order derivatives of $\mathscr {A}$ with respect to *V*, while *μ* is the stationary membrane potential in absence of noise. The functions $\mathscr {B}_{i}\left (t\right )$ are arbitrarily correlated Brownian motions, which represent the source of stochasticity of the model. The model defined in Eq. (1) can be considered a stochastic perturbation to the firing-rate finite-size network model that we previously analyzed in Fasoli et al. (2016).

We define *N*_*E*_ (respectively *N*_*I*_) to be the size of the excitatory (respectively inhibitory) population, with *N*_*E*_ + *N*_*I*_ = *N*, and we rearrange the neurons so that the structural connectivity of the network can be written as follows:

3 $$ J=\left[\begin{array}{cc} \mathfrak{J}_{EE} & \mathfrak{J}_{EI}\\ \mathfrak{J}_{IE} & \mathfrak{J}_{II} \end{array}\right], \qquad \mathfrak{J}_{\alpha\beta}\,=\,\left\{\begin{array}{l} J_{\alpha\alpha}\left( \mathbb{I}_{N_{\alpha}}-\text{Id}_{N_{\alpha}}\right), \;\text{for}\;\alpha=\beta\\ \\ J_{\alpha\beta}\mathbb{I}_{N_{\alpha},N_{\beta}}, \;\text{for}\;\alpha\neq\beta, \end{array}\right.  $$

for *α*,*β* = *E*,*I*. The real numbers *J*_*αβ*_ are free parameters that describe the strength of the synaptic connections from the population *β* to the population *α*. We have *J*_*EE*_,*J*_*IE*_ ≥ 0 and *J*_*EI*_,*J*_*II*_ ≤ 0. Moreover, $\mathbb {I}_{N_{\alpha },N_{\beta }}$ is the *N*_*α*_ × *N*_*β*_ all-ones matrix (here we use the simplified notation $\mathbb {I}_{N_{\alpha }}\overset {\text {def}}{=}\mathbb {I}_{N_{\alpha },N_{\alpha }}$), while $\text {Id}_{N_{\alpha }}$ is the *N*_*α*_ × *N*_*α*_ identity matrix. From our rearrangement of the neurons, we also obtain that the external input currents are organized into two vectors ***I***_*E*,*I*_ such that:

$$\boldsymbol{I}_{\alpha}\left( t\right)=I_{\alpha}\left( t\right)\boldsymbol{1}_{N_{\alpha}}, $$

where $\boldsymbol {1}_{N_{\alpha }}\overset {\text {def}}{=}\mathbb {I}_{N_{\alpha },1}$ is the *N*_*α*_ × 1 all-ones vector. The same subdivision between populations is performed for the parameters *M*, *τ*, *ν* ^max^, Λ, *V* ^*T*^.

We also assume that the covariance structure of the white noise $\sigma _{i}^{\mathscr {B}}\frac {d\mathscr {B}_{i}\left (t\right )}{dt}$ is given by the matrix:

4 $$\begin{array}{@{}rcl@{}} {\Sigma}^{\mathscr{B}} &\,=\,& \left[\begin{array}{cc} \Sigma_{EE}^{\mathscr{B}} & \Sigma_{EI}^{\mathscr{B}}\\ \Sigma_{IE}^{\mathscr{B}} & \Sigma_{II}^{\mathscr{B}} \end{array}\right],\\ \Sigma_{\alpha\beta}^{\mathscr{B}} &\,=\,& \left\{\begin{array}{l} \left( \sigma_{\alpha}^{\mathscr{B}}\right)^{2}\left[\text{Id}_{N_{\alpha}}+C_{\alpha\alpha}^{\mathscr{B}}\left( \mathbb{I}_{N_{\alpha}}-\text{Id}_{N_{\alpha}}\right)\right], \;\text{for}\;\alpha=\beta\\ \sigma_{\alpha}^{\mathscr{B}}\sigma_{\beta}^{\mathscr{B}}C_{\alpha\beta}^{\mathscr{B}}\mathbb{I}_{N_{\alpha},N_{\beta}}, \;\text{for}\;\alpha\neq\beta, \end{array}\right.\\ \end{array} $$

where $C_{\alpha \beta }^{\mathscr {B}}$ are arbitrary parameters that quantify the cross-correlation between the white noise sources. We observe that $C_{EI}^{\mathscr {B}}=C_{IE}^{\mathscr {B}}$ since ${\Sigma }^{\mathscr {B}}$ must be symmetric in order to be a true covariance matrix, and that ${\Sigma }^{\mathscr {B}}$ determines the covariance structure of the white noise since $\text {Cov}\left (\sigma _{i}^{\mathscr {B}}\frac {d\mathscr {B}_{i}\left (t\right )}{dt},\sigma _{j}^{\mathscr {B}}\frac {d\mathscr {B}_{j}\left (s\right )}{ds}\right )=\left [{\Sigma }^{\mathscr {B}}\right ]_{ij}\delta \left (t-s\right )$. Equation (4) represents the most general covariance matrix of the noise under our assumption of fully homogeneous neural populations. Since noise correlations can be interpreted as the amount of shared inputs between nearby neurons (Renart et al., 2010), which is not uniform in the brain, we perform our study for all the possible values of noise correlation, ranging from zero (independent stimuli) to one (identical stimuli to all the neurons).

**Fig. 2:**
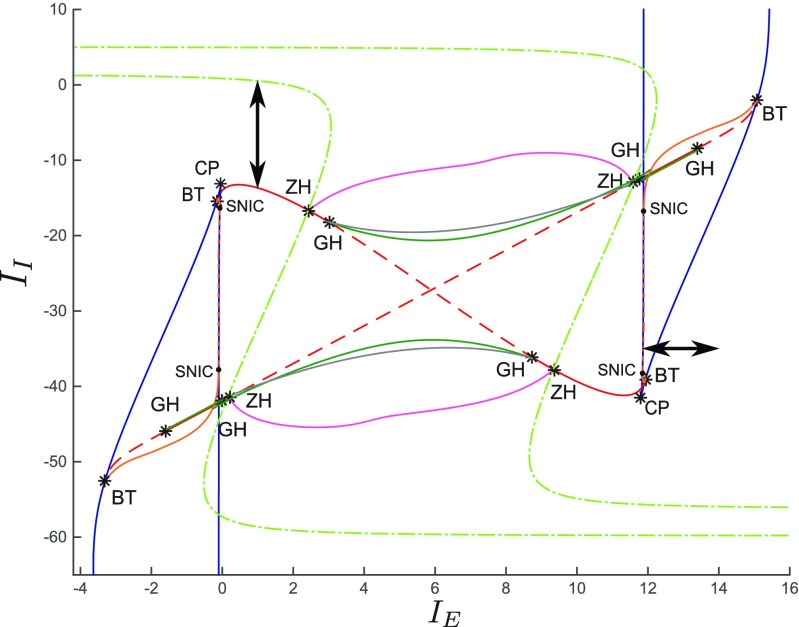
Codimension two bifurcation diagram in the *I*_*E*_ − *I*_*I*_ plane for the two-population network. This diagram was obtained in Fasoli et al. (2016) for the values of the parameters reported in Table 1. The blue curves represent the saddle-node bifurcations (LP for short in Figs. 3, 5) on the primary branch of stationary solutions of Eq. (1), with cusp bifurcations (CP). The red curves correspond to the Andronov-Hopf bifurcations (H for short in Figs. 4, 5) on the primary branch, which in turn are divided into supercritical (plain) and subcritical (dashed) portions. The supercritical/subcritical portions are bounded by a generalized Hopf bifurcation (GH), and Bogdanov-Takens bifurcations (BT). The latter are the contact points among saddle-node, Andronov-Hopf and homoclinic bifurcation curves on the primary branch (hyperbolic-saddle/saddle-node homoclinic bifurcations are represented by plain/dashed orange curves). Saddle-node on invariant circle bifurcations (SNIC) correspond to the contact points between the saddle-node and the homoclinic curves. GH generates limit point of cycles curves, represented by dark green lines, that collapse into the homoclinic curves. The gray lines represent the torus bifurcations, while the light green dot-dashed curves correspond to the branching-point bifurcations (BP for short in Figs. 3, 4 and 5). The purple curves represent the Andronov-Hopf bifurcations that originate from the secondary branches, which meet the branching-point curves and the other Andronov-Hopf curves at the zero-Hopf bifurcations (ZH). The double-headed black arrows represent the ranges in which we varied the stimuli *I*_*E*,*I*_ in order to study the behavior of the cross-correlation. In more detail, on the horizontal arrow the network switches from an asynchronous state to critical slowing down near a saddle-node bifurcation (see also Fig. 3). Moreover, on the vertical arrow the network switches from positively correlated activity at the Andronov-Hopf bifurcation curve, to anti-correlated activity in the inhibitory population at the branching-point curve (see also Fig. 4). Adapted from Fasoli et al. (2016) with permission of the authors

Since we study the case of two neural populations, we can take advantage of the detailed bifurcation analysis performed in Fasoli et al. (2016) (see also Fig. 2), which we will use to determine where the cross-correlation undergoes the most interesting variations.

### Cross-correlation

To quantify statistical dependencies among neurons, we use Pearson cross-correlation, the simplest and most used measure of functional connectivity (David et al., 2004):

5 $$ \text{Corr}\left( V_{i}\left( t\right),V_{j}\left( t\right)\right)\overset{\text{def}}{=}\frac{\text{Cov}\left( V_{i}\left( t\right),V_{j}\left( t\right)\right)}{\sqrt{\text{Var}\left( V_{i}\left( t\right)\right)\text{Var}\left( V_{j}\left( t\right)\right)}}, $$

where Var(*V*_*i*_(*t*)) = Cov(*V*_*i*_(*t*),*V*_*i*_(*t*)). By applying linear response theory around a stable fixed point of the dynamics, we obtain the following analytical expression of the covariance structure of the rate model (1) at the first perturbative order in $\sigma _{i}^{\mathscr {B}}$:

6 $$ \text{Cov}\left( V_{i}\left( t\right),V_{j}\left( t\right)\right)=\sum\limits_{k,l = 0}^{N-1}\left[{\Sigma}^{\mathscr{B}}\right]_{kl}{{\int}_{0}^{t}}\Phi_{ik}\left( t-s\right)\Phi_{jl}\left( t-s\right)ds. $$

In Eq. (6), the matrix ${\Sigma }^{\mathscr {B}}$ is given by Eq. (4), $\Phi \left (t\right )=e^{\mathcal {J}t}$ is the fundamental matrix of the system at time *t*, and $\mathcal {J}$ is its Jacobian matrix (which depends on *J*). Note that this linearized equation is equivalent to that derived in Risken & Frank (1996) (see Eq. (3.45) therein) for a set of linearly coupled Ornstein-Uhlenbeck processes.

When applied to our connectivity matrix (see Eq. (3)), Eqs. (5) and (6) provide a very cumbersome expression of the cross-correlation. Thus, for simplicity, in this article we consider only the infinite-time limit *t* → + *∞* (i.e. the stationary state of the probability distribution of the membrane potentials), even if correlations may be calculated at any finite *t*, if desired.

It is also important to note that under our weak-noise assumption, as proven in Fasoli et al. (2015), the correlations between membrane potentials are equivalent to the correlations between the firing rates of the neurons (which are the quantities normally measured and computed by neuroscientists), as summarized by the following equation:

7 $$ \text{Corr}\left( \nu_{i}\left( t\right),\nu_{j}\left( t\right)\right)\approx\text{Corr}\left( V_{i}\left( t\right),V_{j}\left( t\right)\right). $$

Thus in our paper we will quantify and plot without loss of generality correlations between membrane potentials, but this measure will reflect also the correlations between the firing rates.

### Numerical simulations

To validate our analytical approach, we compared it with numerical evaluations of the correlations in the same network, and we expressed all the results in simulation units (see Section 3). The numerical results were obtained by integrating the neural equations (1) with the Euler-Maruyama scheme, using the parameters reported in Table 1. We used an integration time step of Δ*t* = 10^− 3^ (in simulation units), and the equations were integrated with a Monte Carlo method over 5,000 repetitions of the network dynamics in the temporal interval *t* = [0,30]. Cross-correlations reach a stationary solution after a time period of the order of $\frac {1}{\underset {i = 0,\ldots ,N-1}{\min }\left |\mathfrak {R}\left (\lambda _{i}\right )\right |}$, where $\mathfrak {R}\left (\lambda _{i}\right )$ is the real part of the *i* th eigenvalues of $\mathcal {J}$. Whenever the network is close to a local bifurcation, the real part of (at least) one of its eigenvalues goes to zero, therefore the duration of the transient regime is determined by that eigenvalue. For this reason, the temporal evolution of the cross-correlations slows down. We assumed that at *t* = 30 the transient regime of the correlation has already passed (so that the correlation has already converged to its equilibrium solution), an assumption confirmed a posteriori by the good agreement between the analytical and numerical results.

**Table 1:** Values of the parameters of the two-population network

Population Sizes	Synaptic Weights
and Memb. Time Consts.	
*N*_*E*_ = 8	*J*_*EE*_ = 10
*N*_*I*_ = 2	*J*_*EI*_ = − 70
*τ*_*E*_ = *τ*_*I*_ = 1	*J*_*IE*_ = 70
	*J*_*II*_ = − 34
Activation Functions	Brownian Motions
$\nu _{E}^{\max }=\nu _{I}^{\max }= 1$	$\sigma _{E}^{\mathscr {B}}=\sigma _{I}^{\mathscr {B}}= 10^{-4}$
Λ_*E*_ = Λ_*I*_ = 2	$C_{EE}^{\mathscr {B}}=C_{II}^{\mathscr {B}}=C_{EI}^{\mathscr {B}}= 0$
${V_{E}^{T}}={V_{I}^{T}}= 2$	

We used these parameters to generate all the figures in the article, apart from Figs. 5 and 6 where the cross-correlation was evaluated for different values of $C_{\alpha \beta }^{\mathscr {B}}$. The ratio *N*_*E*_/*N*_*I*_ = 4 matches the proportion between excitatory and inhibitory neurons in real cortical circuits (see Markram et al., 2004). Our theory can be applied to networks of arbitrary size, but in the article we analyze only the case of small networks (*N* = 10 in this example), see text

To conclude, we observe that the numerical integration schemes generally display a loss of stability at the bifurcation points of the network. For this reason, we stabilized the Euler-Maruyama scheme by choosing a small noise amplitude, $\sigma _{E}^{\mathscr {B}}=\sigma _{I}^{\mathscr {B}}= 10^{-4}$.

## Results

In this section we explicitly calculate the cross-correlation structure of the firing-rate network model introduced in Section 2.1, and we study how it depends on the strength of the external stimulus. For simplicity, throughout the main text we consider only the case of two neural populations (for a Python implementation, see the Online Resource 3), but we report the theory for an arbitrary number of populations in the Online Resource 1 and Online Resource 2.

The usefulness and novelty of our approach consists in a convenient decomposition of the spectrum of the Jacobian matrix into “intra-population” eigenvalues, which are generated by the fully-connected architecture within each neural population, and “inter-population” eigenvalues, which depend on the synaptic connections among the populations. According to Eq. (6), the cross-correlations depend on the fundamental matrix of the network, $\Phi \left (t\right )=e^{\mathcal {J}t}$. We define *μ*_*E*,*I*_ to be the stationary membrane potentials in the two populations in absence of noise, namely the zeroth-order approximation to the mean membrane potentials of the stochastic network in the stationary regime. In the Online Resource 1 (see Eq. (S28)) we calculated Φ(*t*) in terms of the intra-population eigenvalues *λ*_*E*,*I*_ and the inter-population eigenvalues $\lambda _{0,1}^{\mathcal {R}}$ of the Jacobian matrix $\mathcal {J}$, evaluated at the stationary solutions:

8 $$\begin{array}{@{}rcl@{}} \lambda_{E}&=&-\left[\frac{1}{\tau_{E}}+\frac{J_{EE}}{M_{E}}\mathscr{A}_{E}^{\prime}\left( \mu_{E}\right)\right],\\ \lambda_{I} &=&-\left[\frac{1}{\tau_{I}}+\frac{J_{II}}{M_{I}}\mathscr{A}_{I}^{\prime}\left( \mu_{I}\right)\right],\\ \lambda_{0,1}^{\mathcal{R}} &=&\frac{\mathcal{Y}+\mathcal{Z}\pm\sqrt{\left( \mathcal{Y}-\mathcal{Z}\right)^{2}+ 4\mathcal{X}}}{2}, \end{array} $$

where:

9 $$\begin{array}{@{}rcl@{}} \mathcal{X}&=&\frac{N_{E}N_{I}}{M_{E}M_{I}}J_{EI}J_{IE}\mathscr{A}_{E}^{\prime}\left( \mu_{E}\right)\mathscr{A}_{I}^{\prime}\left( \mu_{I}\right),\\ \mathcal{Y}&=&-\frac{1}{\tau_{E}}+\frac{N_{E}-1}{M_{E}}J_{EE}\mathscr{A}_{E}^{\prime}\left( \mu_{E}\right),\\ \mathcal{Z}&=&-\frac{1}{\tau_{I}}+\frac{N_{I}-1}{M_{I}}J_{II}\mathscr{A}_{I}^{\prime}\left( \mu_{I}\right), \end{array} $$

and in terms of the functions:

10 $$\begin{array}{@{}rcl@{}} K_{x}\!&\overset{\text{def}}{=}&\!\frac{M_{E}\left( \lambda_{x}^{\mathcal{R}}+\frac{1}{\tau_{E}}\right)-\left( N_{E}-1\right)J_{EE}\mathscr{A}_{E}^{\prime}\left( \mu_{E}\right)}{N_{I}J_{EI}\mathscr{A}_{I}^{\prime}\left( \mu_{I}\right)}\\ &\,=\,&\frac{N_{E}J_{IE}\mathscr{A}_{E}^{\prime}\left( \mu_{E}\right)}{M_{I}\left( \lambda_{x}^{\mathcal{R}}+\frac{1}{\tau_{I}}\right)\,-\,\left( N_{I}\!-1\right)J_{II}\mathscr{A}_{I}^{\prime}\left( \mu_{I}\right)},\quad x\,=\,0,1.\\ \end{array} $$

If the parameters of the network are such that *λ*_*E*,*I*_ and $\lambda _{0,1}^{\mathcal {R}}$ have negative real part, by applying Eqs. (6) and (S28) we obtain the following expression of the covariance matrix of the membrane potentials:

11 $$ {\Sigma}^{V}\!\overset{\text{def}}{=}\!\underset{t\rightarrow+\infty}{\lim}\left[\text{Cov}\left( V_{i}\left( t\right),V_{j}\left( t\right)\right)\right]_{\forall i,j}\,=\,\left[\begin{array}{cc} \Sigma_{EE}^{V} & \Sigma_{EI}^{V}\\ \left[\Sigma{}_{EI}^{V}\right]^{T} & \Sigma_{II}^{V} \end{array}\right]\!, $$

where *T* denotes the transpose of a matrix, while the blocks $\Sigma _{\alpha \beta }^{V}$ are given by the following formulas:

12 $$\begin{array}{@{}rcl@{}} \Sigma_{\alpha\alpha}^{V}&=& \left( \sigma_{\alpha}^{V}\right)^{2}\text{Id}{}_{N_{\alpha}}+\sigma_{\alpha\alpha}^{V}\left( \mathbb{I}_{N_{\alpha}}-\text{Id}{}_{N_{\alpha}}\right),\\ \\ \Sigma_{EI}^{V} &=& \sigma_{EI}^{V}\mathbb{I}_{N_{E},N_{I}}, \\ \\ \left( {\sigma_{E}^{V}}\right)^{2} &=& \left( \sigma_{E}^{\mathscr{B}}\right)^{2}\left[\Upsilon_{EE}^{EE}\Delta_{E}-\left( 1-\frac{1}{N_{E}}\right)\Theta_{E}\right] \\ &&+\left( \sigma_{I}^{\mathscr{B}}\right)^{2}\Upsilon_{EE}^{II}\Delta_{I}+ 2\sigma_{E}^{\mathscr{B}}\sigma_{I}^{\mathscr{B}}\Upsilon_{EE}^{EI}C_{EI}^{\mathscr{B}}, \\ \\ \left( {\sigma_{I}^{V}}\right)^{2}&=& \left( \sigma_{I}^{\mathscr{B}}\right)^{2}\left[\Upsilon_{II}^{II}\Delta_{I}-\left( 1-\frac{1}{N_{I}}\right)\Theta_{I}\right] \\ &&+\left( \sigma_{E}^{\mathscr{B}}\right)^{2}\Upsilon_{II}^{EE}\Delta_{E}+ 2\sigma_{E}^{\mathscr{B}}\sigma_{I}^{\mathscr{B}}\Upsilon_{II}^{EI}C_{EI}^{\mathscr{B}}, \\ \\ \sigma_{EE}^{V}&=& \left( \sigma_{E}^{\mathscr{B}}\right)^{2}\left( \Upsilon_{EE}^{EE}\Delta_{E}+\frac{\Theta_{E}}{N_{E}}\right) \\ &&+\left( \sigma_{I}^{\mathscr{B}}\right)^{2}\Upsilon_{EE}^{II}\Delta_{I}+ 2\sigma_{E}^{\mathscr{B}}\sigma_{I}^{\mathscr{B}}\Upsilon_{EE}^{EI}C_{EI}^{\mathscr{B}}, \\ \\ \sigma_{II}^{V} &=& \left( \sigma_{I}^{\mathscr{B}}\right)^{2}\left( \Upsilon_{II}^{II}\Delta_{I}+\frac{\Theta_{I}}{N_{I}}\right) \\ &&+\left( \sigma_{E}^{\mathscr{B}}\right)^{2}\Upsilon_{II}^{EE}\Delta_{E}+ 2\sigma_{E}^{\mathscr{B}}\sigma_{I}^{\mathscr{B}}\Upsilon_{II}^{EI}C_{EI}^{\mathscr{B}}, \\ \\ \sigma_{EI}^{V} &=& \left( \sigma_{E}^{\mathscr{B}}\right)^{2}\Upsilon_{EI}^{EE}\Delta_{E}+\left( \sigma_{I}^{\mathscr{B}}\right)^{2}\Upsilon_{EI}^{II}\Delta_{I} \\ &&+\sigma_{E}^{\mathscr{B}}\sigma_{I}^{\mathscr{B}}\Upsilon_{EI}^{EI}C_{EI}^{\mathscr{B}}, \\ \\ \Delta_{\alpha}& = & \frac{1}{N_{\alpha}}+C_{\alpha\alpha}^{\mathscr{B}}\left( 1-\frac{1}{N_{\alpha}}\right), \\ \\ \Theta_{\alpha}& = & \frac{1}{2\lambda_{\alpha}}\left( 1-C_{\alpha\alpha}^{\mathscr{B}}\right). \end{array} $$

The functions Υ are defined as below:

$$\begin{array}{@{}rcl@{}} \Upsilon_{EE}^{EE}& = & \frac{1}{\left( K_{1}-K_{0}\right)^{2}}\left[\frac{2K_{0}K_{1}}{\lambda_{0}^{\mathcal{R}}+\lambda_{1}^{\mathcal{R}}}-\frac{1}{2}\left( \frac{{K_{0}^{2}}}{\lambda_{1}^{\mathcal{R}}}+\frac{{K_{1}^{2}}}{\lambda_{0}^{\mathcal{R}}}\right)\right],\\ \Upsilon_{EE}^{II}& = & \frac{1}{\left( K_{1}-K_{0}\right)^{2}}\left[\frac{2}{\lambda_{0}^{\mathcal{R}}+\lambda_{1}^{\mathcal{R}}}-\frac{1}{2}\left( \frac{1}{\lambda_{0}^{\mathcal{R}}}+\frac{1}{\lambda_{1}^{\mathcal{R}}}\right)\right],\\ \Upsilon_{II}^{EE}& = & \frac{{K_{0}^{2}}{K_{1}^{2}}}{\left( K_{1}-K_{0}\right)^{2}}\left[\frac{2}{\lambda_{0}^{\mathcal{R}}+\lambda_{1}^{\mathcal{R}}}-\frac{1}{2}\left( \frac{1}{\lambda_{0}^{\mathcal{R}}}+\frac{1}{\lambda_{1}^{\mathcal{R}}}\right)\right],\\ \Upsilon_{II}^{II}& = & \frac{1}{\left( K_{1}-K_{0}\right)^{2}}\left[\frac{2K_{0}K_{1}}{\lambda_{0}^{\mathcal{R}}+\lambda_{1}^{\mathcal{R}}}-\frac{1}{2}\left( \frac{{K_{0}^{2}}}{\lambda_{0}^{\mathcal{R}}}+\frac{{K_{1}^{2}}}{\lambda_{1}^{\mathcal{R}}}\right)\right],\\ \Upsilon_{EE}^{EI}& = & \frac{1}{\left( K_{1}-K_{0}\right)^{2}}\left[\frac{1}{2}\left( \frac{K_{0}}{\lambda_{1}^{\mathcal{R}}}+\frac{K_{1}}{\lambda_{0}^{\mathcal{R}}}\right)-\frac{K_{0}+K_{1}}{\lambda_{0}^{\mathcal{R}}+\lambda_{1}^{\mathcal{R}}}\right], \end{array} $$

13 $$\begin{array}{@{}rcl@{}} \Upsilon_{II}^{EI}& = & \frac{K_{0}K_{1}}{\left( K_{1}-K_{0}\right)^{2}}\left[\frac{1}{2}\left( \frac{K_{0}}{\lambda_{0}^{\mathcal{R}}}+\frac{K_{1}}{\lambda_{1}^{\mathcal{R}}}\right)-\frac{K_{0}+K_{1}}{\lambda_{0}^{\mathcal{R}}+\lambda_{1}^{\mathcal{R}}}\right], \\ \\ \Upsilon_{EI}^{EE}& = & \frac{K_{0}K_{1}}{\left( K_{1}-K_{0}\right)^{2}}\left[\frac{K_{0}+K_{1}}{\lambda_{0}^{\mathcal{R}}+\lambda_{1}^{\mathcal{R}}}-\frac{1}{2}\left( \frac{K_{0}}{\lambda_{1}^{\mathcal{R}}}+\frac{K_{1}}{\lambda_{0}^{\mathcal{R}}}\right)\right],\\ \Upsilon_{EI}^{II}& = & \frac{1}{\left( K_{1}-K_{0}\right)^{2}}\left[\frac{K_{0}+K_{1}}{\lambda_{0}^{\mathcal{R}}+\lambda_{1}^{\mathcal{R}}}-\frac{1}{2}\left( \frac{K_{0}}{\lambda_{0}^{\mathcal{R}}}+\frac{K_{1}}{\lambda_{1}^{\mathcal{R}}}\right)\right], \\ \\ \Upsilon_{EI}^{EI}& = & \frac{1}{\left( K_{1}-K_{0}\right)^{2}}\left[K_{0}K_{1}\left( \frac{1}{\lambda_{1}^{\mathcal{R}}}+\frac{1}{\lambda_{0}^{\mathcal{R}}}-\frac{2}{\lambda_{0}^{\mathcal{R}}+\lambda_{1}^{\mathcal{R}}}\right)\right. \\ &&\left.-\frac{{K_{0}^{2}}+{K_{1}^{2}}}{\lambda_{0}^{\mathcal{R}}+\lambda_{1}^{\mathcal{R}}}\right]. \end{array} $$

In Eq. (12), $\sigma _{\alpha }^{V}$ is the standard deviation of the membrane potentials in the population *α*, while $\sigma _{\alpha \alpha }^{V}$ is the covariance between any pair of potentials in the same population *α*, and $\sigma _{EI}^{V}$ is the covariance between any pair of potentials in two different populations.

From the above equations, the correlation matrix is obtained simply by normalizing the covariance matrix (11) as follows (see Eq. (5)). In particular, we call $C_{\alpha \beta }^{V}$:

$$C_{\alpha\beta}^{V}\overset{\text{def}}{=}\frac{\sigma_{\alpha\beta}^{V}}{\sigma_{\alpha}^{V}\sigma_{\beta}^{V}} $$

the entries of the correlation matrix that represent the correlation between any pair of membrane potentials in populations *α*,*β*.

In the following sections we will study how the above obtained analytical expression of the cross-correlation varies when changing the network parameters. This will be used to show that, depending on the values of the parameters, the network can switch from asynchronous to synchronous states. These regimes have radically contrasting properties, which will be discussed in detail in Sections 3.1 and 3.2.

### Asynchronous states

We will first examine the existence of network states characterized by small cross-neuron correlations. Networks are said to be in an *asynchronous regime* when they show uncorrelated activity (Ecker et al., 2010; Renart et al., 2010; Tetzlaff et al., 2012; Grytskyy et al., 2013). In mathematics, a regime characterized by statistically independent (though interacting) units is called *local chaos* (Boltzmann 1872; Samuelides & Cessac 2007; Touboul et al., 2012; Baladron et al., 2012; Fasoli et al., 2015). Note that however in our model the asynchronous state and local chaos are equivalent at the first perturbative order, since neurons are jointly normally distributed under our weak-noise assumption.

The characterizing feature of an asynchronous state is the weak cross-correlations between the membrane potentials. Moreover, in our model asynchrony generally occurs with small fluctuations of the potentials, as we prove below. The most known and straightforward way to generate an asynchronous state with negligible correlations across neurons is using a network of large or infinite size (Ginzburg & Sompolinsky 1994; Samuelides & Cessac 2007; Touboul et al., 2012; Baladron et al., 2012; Baladron Pezoa et al., 2012). Indeed, if $C_{EE}^{\mathscr {B}}=C_{II}^{\mathscr {B}}=C_{EI}^{\mathscr {B}}= 0$, from Eqs. (12) and (13) it follows that $\sigma _{\alpha \beta }^{V}\rightarrow 0$ and $\left (\sigma _{\alpha }^{V}\right )^{2}\rightarrow \left (-\frac {1}{2\lambda _{\alpha }}\right )\left (\sigma _{\alpha }^{\mathscr {B}}\right )^{2}\approx \frac {1}{2\tau _{\alpha }}\left (\sigma _{\alpha }^{\mathscr {B}}\right )^{2}$ (for *α*,*β* = *E*,*I*) in the thermodynamic limit *N*_*E*,*I*_ →*∞*. In other words, in infinite-size networks with independent Brownian motions, the membrane potentials are independent too, leading to local chaos with small fluctuations (indeed, under our weak-noise assumption, their standard deviation $\sigma _{\alpha }^{V}\approx \frac {\sigma _{\alpha }^{\mathscr {B}}}{\sqrt {2\tau _{\alpha }}}$ is small). Local chaos is usually invoked to justify the mean-field description of large neural networks and is compatible with recent findings in visual cortex (Ecker et al., 2010; Renart et al., 2010; Tetzlaff et al., 2012).

Interestingly, also finite-size networks can however experience decorrelated activity. In Fasoli et al. (2015) the authors showed that, for any *N*, weak correlations occur for strongly depolarizing or strongly hyperpolarizing external inputs, if the Brownian motions are independent. This phenomenon can be proven to occur in networks with any topology from general considerations about its Jacobian matrix (see the Online Resource 1). However, this approach is only qualitative, therefore it does not provide any explicit formula of the cross-correlation. In Fasoli et al. (2015), explicit expressions of the correlation structure were obtained for networks with regular topology, through the analytical calculation of the eigenquantities of the synaptic connectivity matrix. In this work we extended the analytical calculations to multi-population networks (an explicit example is shown in Eqs. (11), (12), (13) for the two-population case), whose topology is generally irregular since the corresponding graph has non-uniform synaptic weights.

**Fig. 3:**
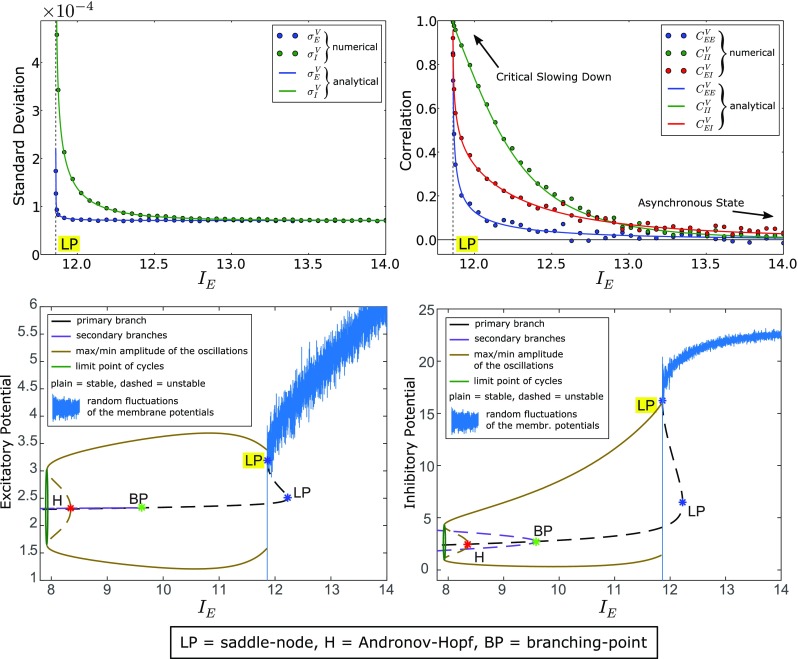
Transition between asynchronous and synchronous states near a saddle-node bifurcation. The top panels show a good agreement between the numerical approximations of the standard deviation and correlation (left and right panel respectively), and the corresponding analytical formulas (see Eqs. (12) and (13)). The numerical approximations have been obtained through the methods described in Section 2.3. For large inputs (*I*_*E*_ > 13, see also Fig. 2) we observe the formation of an asynchronous state, which is characterized by weak correlation and low variability. On the other hand, near a saddle-node bifurcation (*I*_*E*_ ≈ 11.86, see the highlighted LP), we observe the formation of a synchronous state characterized by strong correlations and wide temporal fluctuations (critical slowing down). The bottom panels show numerical simulations of the fluctuations of the membrane potentials in the excitatory and inhibitory population (left and right panel respectively), calculated at *t* = 30 for different values of *I*_*E*_ and superposed to the codimension one bifurcation diagram of the network. The fluctuations are displayed at 3,000 × actual size in the excitatory and inhibitory population, in order to make them visible on the bifurcation diagrams. The reader may verify the agreement between the standard deviations (top-left panel) and the envelope of the fluctuations of the membrane potentials

By taking advantage of the analytical expressions of the cross-correlation, we observe that the formation of asynchronous states can be proven for the two-population case as a consequence of $\Upsilon _{\alpha \alpha }^{\alpha \alpha }\rightarrow -\frac {1}{2\lambda _{\alpha }}$, $\Upsilon _{\beta \beta }^{\alpha \alpha }\rightarrow 0$ (with *α*≠*β*) and $\Upsilon _{EI}^{\alpha \alpha }\rightarrow 0$ for |*I*_*E*,*I*_| →*∞*, which in turn is due to the saturation of the activation function $\mathscr {A}\left (V\right )$. For the same reason, the standard deviations $\sigma _{E,I}^{V}$ of the membrane potentials in the two populations decrease with the input. Interestingly, the input-driven reduction of both the correlation and the variance of the neural responses is supported by experimental evidence (Tan et al., 2014; Ponce-Alvarez et al., 2015). Figure 3 shows an example of formation of an asynchronous regime, which is obtained for the values of the parameters in Table 1 and for strong stimuli (*I*_*E*_ > 13, *I*_*I*_ = − 35).

The theory developed in this article can also be applied when the noise sources in Eq. (1) are correlated. However, given arbitrary (i.e. non-specifically tuned) parameters, the firing-rate network model of Eq. (1) generally does not undergo the formation of asynchronous states when the Brownian motions are not independent. In particular, since $\Upsilon _{\alpha \alpha }^{EI}\rightarrow 0$ and $\Upsilon _{EI}^{EI}\rightarrow \frac {1}{2\sqrt {\lambda _{E}\lambda _{I}}}$ for |*I*_*E*,*I*_| →*∞*, we get $\left (\sigma _{\alpha }^{V}\right )^{2}\rightarrow \frac {1}{2\tau _{\alpha }}\left (\sigma _{\alpha }^{\mathscr {B}}\right )^{2}$ and $C_{\alpha \beta }^{V}\rightarrow C_{\alpha \beta }^{\mathscr {B}}$ (see also Figs. 5 and 6, where we plot the cross-correlations for different values of $C^{\mathscr {B}}$). In other words, in the considered small-size firing-rate model, for strong stimuli the correlation between the membrane potentials converges to that between the Brownian motions, and again the fluctuations of the membrane potentials have small standard deviation. Note, however, that other network models (such as the networks of non-leaky integrate-and-fire neurons considered in Moreno-Bote 2014 and Moreno-Bote et al., 2014) can have very weak correlations even for correlated inputs if the connectivity matrix is specifically tuned for that.

**Fig. 4:**
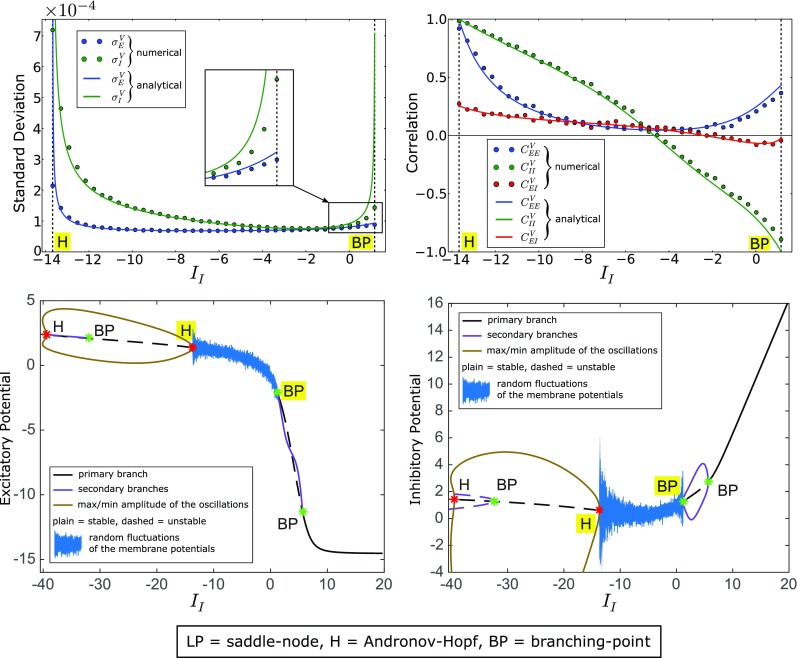
Fluctuations and cross-correlations of the membrane potentials between Andronov-Hopf and branching-point bifurcations. The simulations are similar to those of Fig. 3), but now we set *I*_*E*_ = 1 and we vary the input to the inhibitory population (see Fig. 2), obtaining a transition between an Andronov-Hopf bifurcation (*I*_*I*_ ≈− 13.67, see the highlighted H) and a branching-point bifurcation (*I*_*I*_ ≈ 1.165, highlighted BP). We obtain a good agreement between numerical and analytical correlations for any current *I*_*I*_ in the range, while the standard deviations display a good agreement only when *I*_*I*_ is sufficiently far from the bifurcation points. At the Andronov-Hopf and branching-point bifurcations the standard deviations predicted by the analytical formulas are larger than those obtained numerically. This suggests that generally second-order corrections to Eqs. (12) and (13) play a stronger role when the network undergoes these local bifurcations. Nevertheless, the first-order approximation describes qualitatively the increase of the standard deviation that characterizes critical slowing down

**Fig. 5:**
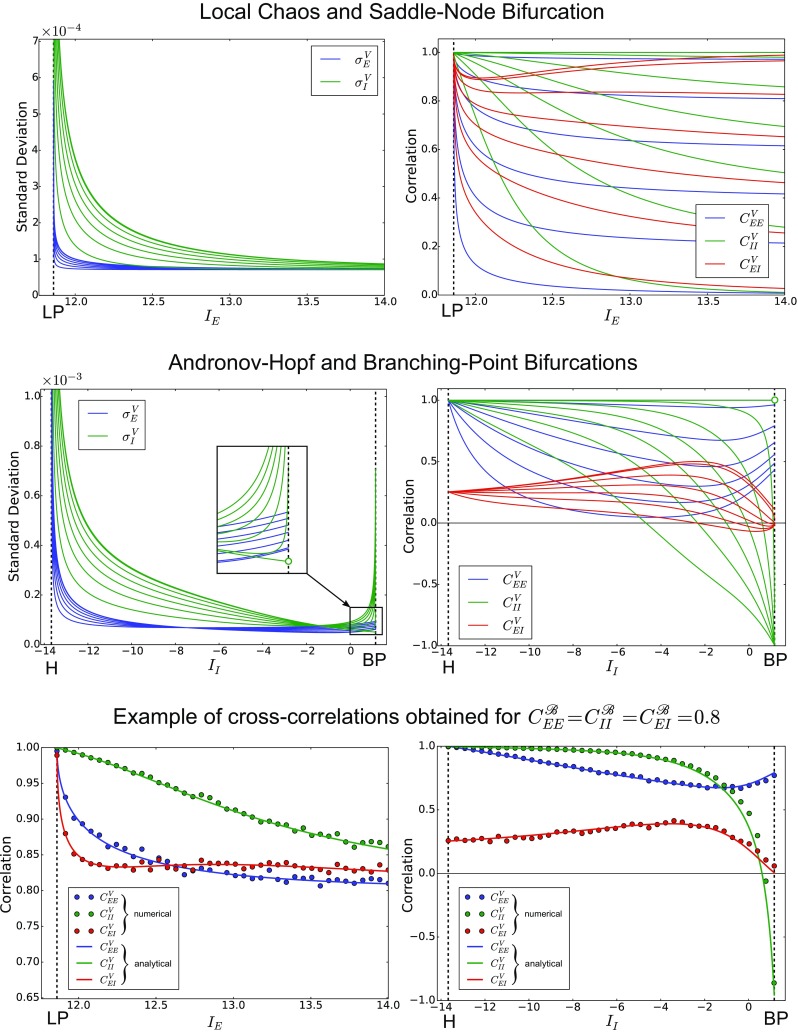
Fluctuations and cross-correlations of the membrane potentials as a function of the input and of the noise correlation. The top panels show the standard deviation (left) and the cross-correlation (right) of the membrane potentials when the network is close to a saddle-node bifurcation (similarly to Fig. 3), for different values of the noise correlation. The curves have been obtained from Eqs. (12) and (13) for $C_{EE}^{\mathscr {B}}=C_{II}^{\mathscr {B}}=C_{EI}^{\mathscr {B}}= 0,\,0.2,\,0.4,\,0.6,\,0.8,\,0.97,\,1$. The panels show that the noise correlation increases both the standard deviation and the cross-correlation, for $\sigma _{E,I}^{\mathscr {B}}$ fixed (see Table 1). The middle panels show similar results for the neural states between Andronov-Hopf and branching-point bifurcations (compare with Fig. 4). The only difference is observed close to the branching-point bifurcation, where *σ* *E*,*I* *V* decrease with the noise correlation. The bottom panels show the comparison between the analytical and numerical cross-correlations in the case $C_{EE}^{\mathscr {B}}=C_{II}^{\mathscr {B}}=C_{EI}^{\mathscr {B}}= 0.8$

**Fig. 6:**
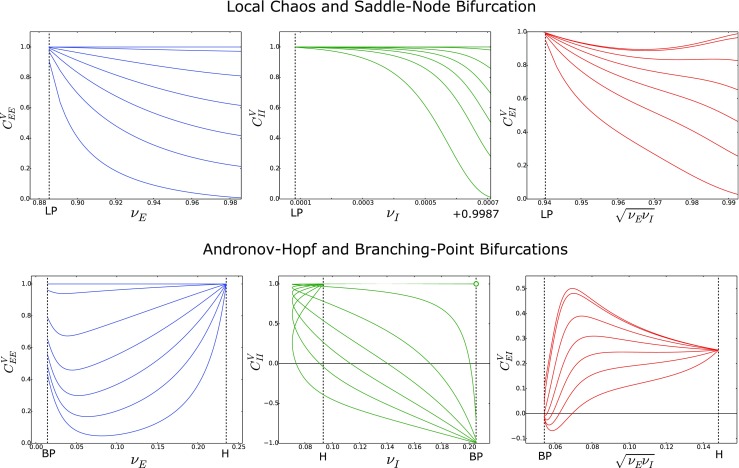
Cross-correlations of the membrane potentials as a function of the firing rate and of the noise correlation. The figure shows the cross-correlation of the membrane potentials as a function of the geometric mean of the firing rates $\sqrt {\nu _{\alpha }\nu _{\beta }}=\sqrt {\mathscr {A}_{\alpha }\left (\mu _{\alpha }\right )\mathscr {A}_{\beta }\left (\mu _{\beta }\right )}$ for *α*,*β* = *E*,*I*. Similarly to Fig. 5, the curves have been obtained from Eqs. (12) and (13) for $C_{EE}^{\mathscr {B}}=C_{II}^{\mathscr {B}}=C_{EI}^{\mathscr {B}}= 0,\,0.2,\,0.4,\,0.6,\,0.8,\,0.97,\,1$. The panels show a non-monotonic dependence of the correlation on the firing rates

### Synchronous states

We now examine the existence of network states characterized by large correlations among neurons, called *synchronous states* (Ginzburg & Sompolinsky 1994; De La Rocha et al., 2007; Harris & Thiele 2011).

Large correlations can be generated in two different ways. The most straightforward is by increasing the correlation between the Brownian motions, while a more subtle one takes advantage of the phenomenon called *critical slowing down* (Scheffer et al., 2009; Kéfi et al., 2013). Contrary to asynchronous states, the most important features of critical slowing down are large temporal fluctuations of the membrane potentials and strong cross-correlations (even though the Brownian motions are independent). Critical slowing down typically occurs at the bifurcation points of the system. Near local bifurcations, the real part of one of the eigenvalues tends to zero, therefore the system becomes increasingly slow in recovering from small perturbations. As a consequence, the system has a longer memory for perturbations, and its dynamics is characterized by larger stochastic fluctuations and stronger correlations. In Fasoli et al. (2016) we performed a detailed bifurcation analysis in the two-population case and for the values of the parameters in Table 1, obtaining the entangled set of local and global bifurcations shown in Fig. 2. Local bifurcations occur when a parameter variation causes the stability of an equilibrium point to change, therefore they are studied through the eigenvalues of the Jacobian matrix. Local bifurcations can be of codimension one or two, depending on the number of parameters (e.g. *I*_*E*,*I*_) that must be varied for the bifurcation to occur. As shown in Fig. 2, the local bifurcations of codimension one the network undergoes are saddle-node, Andronov-Hopf and branching-point bifurcations, while those of codimension two are cusp, Bogdanov-Takens, generalized Hopf and zero-Hopf bifurcations. As discussed in Fasoli et al. (2016), for *N*_*I*_ > 2 other kinds of local bifurcations of codimension two may occur due to spontaneous-symmetry breaking (e.g. the double-Hopf bifurcation). Nevertheless, for simplicity, in the main text we restrict our discussion to the case *N*_*I*_ = 2.

Similarly to Kuehn (2013), we study the behavior of the correlation only at the local bifurcations of the network, and in particular we consider only those of codimension one. These bifurcations are studied in the following paragraphs for the case of two neural populations, and in Section (S6) of the Online Resource 1 for the case of an arbitrary number of populations. Our theory can also be used to study the behavior of the correlation near local bifurcations of codimension two, but due to the high variety of the bifurcations the system exhibits, a complete study is beyond the purpose of this article. We note that there are also global bifurcations (for example the homoclinic, limit point of cycles, and torus^1^ curves that are global bifurcations of codimension one, and saddle-node on invariant circle points that represent the only global bifurcations of codimension two). However, to our knowledge no analytical method is known for studying the global bifurcations of Eq. (1). We therefore restrict our analysis to local bifurcations only.

#### Saddle-node bifurcations (catastrophic transitions)

Saddle-node bifurcations occur in many dynamical systems. They represent tipping points at which tiny perturbations can cause an abrupt and discontinuous change of the equilibrium point of the system. In particular, in neuroscience some authors proposed that the whole-cortex activity may undergo saddle-node bifurcations during anesthetic administration at the edge between conscious and unconscious states (Steyn-Ross et al., 2004).

In Fasoli et al. (2016) we proved that, in the two-population case, the network undergoes a saddle-node bifurcation whenever one of the eigenvalues $\lambda _{0,1}^{\mathcal {R}}$ in Eq. (8) tends to zero. The saddle-node bifurcations are described by the blue curves in Fig. 2. In Fasoli et al. (2016) we also proved that a necessary condition for the formation of these bifurcations is:

14 $$ \frac{N_{E}-1}{N-1}J_{EE}\frac{\nu_{E}^{\max}\Lambda_{E}}{4}\tau_{E}>1, $$

or in other words sufficiently strong self-excitatory weights are required. From Eq. (13) we observe that for $\lambda _{0}^{\mathcal {R}}\rightarrow 0^{-}$ or $\lambda _{1}^{\mathcal {R}}\rightarrow 0^{-}$ the functions Υ diverge, therefore the terms proportional to $\frac {1}{2\lambda _{E,I}}$ in Eq. (12) become negligible. This implies $\sigma _{\alpha \alpha }^{V}\sim \left (\sigma _{\alpha }^{V}\right )^{2}\rightarrow \infty $ and $\sigma _{EI}^{V}{\sim \sigma _{E}^{V}}{\sigma _{I}^{V}}$, therefore $C_{\alpha \beta }^{V}\sim 1$ between every population. Thus, when the network is close to a saddle-node bifurcation, we observe the emergence of critical slowing down. Moreover, we obtain a simple relation between the variances of the two neural populations, namely *σ* *I* *V* ∼ *K* *σ* *E* *V*, where $K\overset {\text {def}}{=}\underset {\lambda _{0}^{\mathcal {R}}\rightarrow 0^{-}}{\lim }K_{0}=\underset {\lambda _{1}^{\mathcal {R}}\rightarrow 0^{-}}{\lim }K_{1}=\frac {N_{E}J_{IE}\mathscr {A}_{E}^{\prime }\left (\mu _{E}\right )}{\frac {M_{I}}{\tau _{I}}-\left (N_{I}-1\right )J_{II}\mathscr {A}_{I}^{\prime }\left (\mu _{I}\right )}$. The reader can also verify that $\sigma _{EI}^{V}>0$ for $\lambda _{\alpha }^{\mathcal {R}}\rightarrow 0$ as a consequence of *K* > 0, which in turn is due to *J*_*IE*_ > 0 and *J*_*II*_ < 0. An example of critical slowing down obtained for *I*_*E*_ ≈ 11.86, *I*_*I*_ = − 35 and $C_{EE}^{\mathscr {B}}=C_{II}^{\mathscr {B}}=C_{EI}^{\mathscr {B}}= 0$ is reported in Fig. 3. We observe that this phenomenon occurs even if there is no correlation between the Brownian motions (i.e. $C_{\alpha \beta }^{\mathscr {B}}= 0$), therefore it is entirely a consequence of the neural interactions mediated by the synaptic connections.

#### Andronov-Hopf bifurcations (oscillations)

Andronov-Hopf bifurcations correspond to the emergence of neural oscillations, which are a phenomenon often seen in cortical activity and which is thought to play a key role in many cognitive processes (Ward 2003). In the two-population case, the network undergoes an Andronov-Hopf bifurcation whenever $\lambda _{0,1}^{\mathcal {R}}$ in Eq. (8) are complex-conjugate purely imaginary. The Andronov-Hopf bifurcations are described by the red curves in Fig. 2. In Fasoli et al. (2016) we proved that a necessary condition for the formation of these bifurcations is:

15 $$ \frac{\nu_{E}^{\max}\Lambda_{E}}{4\mathfrak{z}}>1\quad\text{and}\quad\mathfrak{b}^{2}-4\mathfrak{a}\mathfrak{c}>0 $$

where:

$$\begin{array}{@{}rcl@{}} \mathfrak{z}&=&\frac{-\mathfrak{b}-\sqrt{\mathfrak{b}^{2}-4\mathfrak{a}\mathfrak{c}}}{2\mathfrak{a}},\\ \\ \mathfrak{a}&=&\left( \frac{N_{E}-1}{N-1}J_{EE}\right)^{2}-\frac{N_{E}N_{I}\left( N_{E}-1\right)}{\left( N-1\right)^{2}\left( N_{I}-1\right)}\frac{J_{EE}J_{EI}J_{IE}}{J_{II}},\\ \\ \mathfrak{b}&=&-\frac{2}{\tau_{E}}\frac{N_{E}-1}{N-1}J_{EE}+\frac{N_{E}N_{I}}{\left( N\,-\,1\right)\left( N_{I}\,-\,1\right)}\frac{J_{EI}J_{IE}}{J_{II}}\left( \frac{1}{\tau_{E}}\,+\,\frac{1}{\tau_{I}}\right),\\ \\ \mathfrak{c}&=&\frac{1}{{\tau_{E}^{2}}}. \end{array} $$

The mechanism of formation of critical slowing down at the Andronov-Hopf bifurcations is similar to that described in the previous paragraph for the saddle-node-bifurcations, therefore we discuss it only briefly. Whenever the network approaches an Andronov-Hopf bifurcation, we get $\lambda _{0}^{\mathcal {R}}+\lambda _{1}^{\mathcal {R}}\rightarrow 0^{-}$, which causes the terms Υ to diverge (see Eq. (13)). For this reason the variance of the membrane potentials diverges as well, while the cross-correlation tends to one, similarly to the case of the saddle-node bifurcations. This proves that the network undergoes critical slowing down also at the Andronov-Hopf bifurcations. The main difference with the case of the saddle-node-bifurcations is represented by the inter-population correlation $C_{EI}^{V}$, which does not tend to one near the Andronov-Hopf bifurcations (this phenomenon is discussed in more detail in Section (S6.2.2) of the Online Resource 1). An example obtained for *I*_*E*_ = 1, *I*_*I*_ ≈− 13.67 and $C_{EE}^{\mathscr {B}}=C_{II}^{\mathscr {B}}=C_{EI}^{\mathscr {B}}= 0$ is shown in Fig. 4.

#### Branching-point bifurcations (spontaneous symmetry-breaking)

In the deterministic model (i.e. for $\sigma _{\alpha }^{\mathscr {B}}= 0$), because of the homogeneity assumption of Section 2.1, neurons within each population are expected to have identical dynamics. This means that, in the absence of noise, the network dynamics is invariant under transformations in the group $S_{N_{0}}\times \cdots \times S_{N_{\mathfrak {P}-1}}$, where $S_{N_{\alpha }}$ is the permutation group on *N*_*α*_ items (also known as *symmetric group*). When we include noise ($\sigma _{\alpha }^{\mathscr {B}}>0$), we introduce a small explicit symmetry-breaking into Eq. (1). However,the behavior of a nearly symmetric dynamical system is more similar to that of an idealized symmetric system than that of a completely asymmetric one (Stewart et al., 2003). Therefore, if the degree of explicit heterogeneity introduced by the noise is not too strong, it is legitimate to study Eq. (1) as a perturbation of the corresponding deterministic system. However, symmetry-breaking may occur also in the deterministic model. Indeed, at the branching-point bifurcations we observe the formation of a *spontaneous symmetry-breaking* (Fasoli et al., 2016) because some of the neurons within a given inhibitory population become dynamically distinct from the others. In other words, we observe the formation of an heterogeneous inhibitory population, even if the neural equations (1) for $\sigma _{\alpha }^{\mathscr {B}}= 0$ do not contain any term that breaks explicitly the symmetry. Interestingly, this phenomenon is a consequence of the finite size of the network, therefore it does not occur in the thermodynamic limit (Fasoli et al., 2016).

In Fasoli et al. (2016) we also proved that, in the two-population case, branching-point bifurcations occur whenever *λ*_*I*_ = 0 (see the light green dot-dashed curves in Fig. 2) and that a necessary condition for their formation is:

16 $$ \frac{\tau_{I}\left|J_{II}\right|\nu_{I}^{\max}\Lambda_{I}}{4\left( N-1\right)}>1. $$

This means that sufficiently strong self-inhibitory weights are required for the bifurcations to occur. According to Eq. (12), for $C_{II}^{\mathscr {B}}<1$ and *λ*_*I*_ → 0^−^ only the variance of the inhibitory neurons diverges. As a consequence, in the case $C_{II}^{\mathscr {B}}<1$ we get $\left ({\sigma _{I}^{V}}\right )^{2}\sim -\left (\sigma _{I}^{\mathscr {B}}\right )^{2}\left (1-\frac {1}{N_{I}}\right )\Theta _{I}$ and $\sigma _{II}^{V}\sim \left (\sigma _{I}^{\mathscr {B}}\right )^{2}\frac {\Theta _{I}}{N_{I}}$, from which we conclude that $C_{II}^{V}\sim \frac {1}{1-N_{I}}$. According to Fasoli et al. (2015), this is the lower bound of the correlation between fully-connected neurons in a homogeneous population with size *N*_*I*_. Since $\frac {1}{1-N_{I}}<0$ for *N*_*I*_ ≥ 2, at the branching-point bifurcations the inhibitory neurons are maximally anti-correlated (in particular, the correlation tends to − 1 only for *N*_*I*_ = 2). From these results we conclude that, contrary to the saddle-node and Andronov-Hopf bifurcations, at the branching points critical slowing down occurs only in the inhibitory population. This is confirmed by Fig. 4, which shows an example obtained for *I*_*E*_ = 1, *I*_*I*_ ≈ 1.165 and $C_{EE}^{\mathscr {B}}=C_{II}^{\mathscr {B}}=C_{EI}^{\mathscr {B}}= 0$. Intuitively, the membrane potentials become anti-correlated because the inhibitory neurons follow different branches of stationary solutions beyond the branching-point (see the codimension one bifurcation diagram in the bottom-right panel of Fig. 4). Therefore while the potential of one neuron increases due to noise fluctuations, the potential of the other neuron decreases and viceversa, resulting in a negative correlation.

On the other hand, for $C_{II}^{\mathscr {B}}= 1$ and *λ*_*I*_ → 0^−^, from Eq. (12) we get:

$$\begin{array}{@{}rcl@{}} \left( {\sigma_{I}^{V}}\right)^{2}&=&\sigma_{II}^{V}=\left( \sigma_{E}^{\mathscr{B}}\right)^{2}\Upsilon_{II}^{EE}\Delta_{E}+\left( \sigma_{I}^{\mathscr{B}}\right)^{2}\Upsilon_{II}^{II}\\ &&+ 2\sigma_{E}^{\mathscr{B}}\sigma_{I}^{\mathscr{B}}\Upsilon_{II}^{EI}C_{EI}^{\mathscr{B}}, \end{array} $$

therefore now $\left ({\sigma _{I}^{V}}\right )^{2}$ does not diverge anymore and $C_{II}^{V}= 1$ (see also Figs. 5 and 6). To conclude, for $C_{II}^{\mathscr {B}}= 1$ and *λ*_*I*_ = 0, Eq. (12) gives an indeterminate form $\frac {0}{0}$ for the variance $\left ({\sigma _{I}^{V}}\right )^{2}$ and the covariance $\sigma _{II}^{V}$, which is represented by the empty circles in the middle panels of Fig. 5 and in the bottom-central panel of Fig. 6. This result can be intuitively interpreted as the consequence of the competition between the positive correlation introduced by the Brownian motions and the anti-correlation generated by the branching point.

## Discussion

We developed a theory of correlations in a multi-population graded-firing-rate network model of arbitrary size. This theory, taking advantage of mathematical methods (Fasoli et al., 2015; Fasoli et al., 2016) not based on statistical averages, allows a rigorous analytical understanding of how correlations depend on the parameters and structure of networks of arbitrary size. These networks include small-size circuits of a few tens of neurons, such as for example neural circuits in some invertebrates (Williams & Herrup 1988), and are not restricted to large systems as in previous work (Ginzburg & Sompolinsky 1994; Bressloff 2009; Renart et al., 2010; Buice & Chow 2013). Importantly, our formalism allowed us to investigate the interplay between network dynamics and network statistics, by combining the non-linear analysis of the bifurcations of the network with a linear analysis of the covariance matrix.

Through our formalism, explicit calculations of the cross-correlations are possible only for networks composed of a few neural populations. In particular, for exemplary purposes, in the main text we focused on the case of a two-population network, composed of excitatory and inhibitory neurons, and we extensively validated the closed-form expression of its correlation structure through numerical simulations. However, it is important to observe that the more abstract formalism introduced in the Online Resource 1 does not rely on the explicit calculation of the cross-correlations, and therefore predicts the formation of synchronous and asynchronous states in networks composed of an arbitrary number of neural populations.

In the following we discuss the advances of our results with respect to previous work, and the implications of our work to better understand neural network dynamics.

### Progress with respect to previous modeling work

A first advance with respect to some previous theories of correlations based on rate models made of binary neurons (Ginzburg & Sompolinsky 1994; Renart et al., 2010), was that we were able to introduce a biologically more realistic network composed of graded neurons with continuous firing rate, without losing the possibility to derive analytical expressions for the network correlations. However, a more important advance with respect to these previous works was that we could consider correlations among populations of arbitrary size rather than of large size. In the context of neuroscience, this is important because some networks (e.g. the nervous system of some invertebrates such as rotifers and nematodes) are composed only of a few tens of cells (Williams & Herrup 1988), and because small networks have, as shown in this work, different relationships between dynamics and correlations than the large scale ones. In particular, the main difference between small and large networks is the formation of spontaneous-symmetry breaking in the inhibitory populations. This phenomenon, which we discuss in the next subsections, increases considerably the complexity of the dynamics in small networks, and determines relationships between dynamics and correlations that are not predicted by large-network studies.

In the recent work (Fasoli et al., 2015), the authors were able to begin investigating some properties of correlations in small networks. However, in this earlier work, they could only investigate neural circuits with regular topology, while here we extended their theory to multi-population networks with irregular topology. Importantly, and unlike in Fasoli et al. (2015), here we studied the relation between the correlation structure of the network and the bifurcation points of the neural dynamics. Specifically, we studied the behavior of the correlation in terms of the stimuli *I*_*α*_, and this analysis revealed the ability of the network to switch dynamically from asynchronous regimes, characterized by weak correlation and low variability, to synchronous regimes, characterized by strong correlations and wide temporal fluctuations of the state variables.

Note that the mathematical bases for systematically studying critical slowing down at bifurcations up to codimension two were laid out in general terms in Kuehn (2013). Here, we show that such deep mathematical concepts can be realized and found in neural networks.

### New insights into the relation between network dynamics and correlation structure

These mathematical advances with respect to previous work allowed us to reach a set of novel insights into the relation between the correlation structure of the network and the bifurcation points of the neural dynamics. In particular, we found how transitions between synchronous and asynchronous states relate to bifurcations.

The asynchronous regime can be observed in large networks driven by independent sources of noise (Samuelides & Cessac 2007; Touboul et al., 2012; Baladron et al., 2012; Baladron Pezoa et al., 2012). Here, however, we proved that in the small-size firing-rate network model considered here asynchronous states can be generated dynamically by strong stimuli. In the firing-rate model we study, this phenomenon occurs for arbitrary (i.e. not specifically tuned) network parameters, provided the input is strong enough and the Brownian motions are independent. This is a consequence of the saturation of the activation function which avoids blowing-up solutions. However, asynchronous states can occur through this mechanism also in networks with non-saturating functions for specific values of the network parameters. Interestingly, the decrease of both the variance and the cross-correlation of the neural responses with the input is supported by experimental evidence (Tan et al., 2014; Ponce-Alvarez et al., 2015). For arbitrary (i.e. non-specifically tuned) parameters, the small-size firing-rate network model of Eq. (1) in general does not undergo the formation of asynchronous states when the Brownian motions are correlated. However, it is important to note that, unlike our case of small-size circuits, in large neural networks several mechanisms have been shown to be able to decorrelate network activity even in the presence of correlated inputs (see e.g. van Vreeswijk and Sompolinsky 1998; Renart et al., 2010; Tetzlaff et al., 2012; Hennequin et al., 2016).

Importantly, we found that the synchronous regime occurs near the bifurcation points of the network, which in this model can be analytically determined (Fasoli et al., 2016). In particular, in the present article we considered the local bifurcations of codimension one, namely the saddle-node, Andronov-Hopf and branching-point bifurcations. Contrary to the strongly positive correlations that occur at the saddle-node and Andronov-Hopf bifurcations, at the branching points we have observed the emergence of strong *anti-correlations* between inhibitory neurons.

The emergence of strong correlations at any of the local bifurcations of the network is a finite-size effect, and does not require correlated sources of noise. Indeed, for a network with independent Brownian motions, in Fasoli et al. (2015) the authors proved that the neurons are strongly synchronized at a time instant *t*_*N*_ that depends on the size of the network. Strong correlations are very unlikely to occur in large networks after short time intervals, since *t*_*N*_ →*∞* in the limit *N* →*∞*. However, exceptions may arise in sparsely-connected networks (see Section 4.5), or if the Brownian motions are correlated.

It is also important to observe that, in the case of networks made of two populations, Eqs. (11), (12), (13) represent a mathematical description of a multidimensional continuum of states, ranging from asynchronous to synchronous states, and corresponding to varying levels of spontaneous fluctuations and cross-correlation in neural population activity. The existence of this continuum was first proposed in Harris & Thiele (2011), where the authors observed that the multidimensional nature of the continuum emerges from analyzing the structure of the fluctuations and of the cross-correlation under several different behavioral and experimental conditions. These conditions can be effectively modeled for example by varying the strength of the synaptic weights (see Section 4.4), or by varying the strength of the input parameters *I*_*E*,*I*_ in order to describe the presence or absence of external stimulation.

### Spontaneous symmetry-breaking as the origin of anti-correlations

We proved that at the branching-point bifurcations the inhibitory neurons become strongly anti-correlated as a consequence of spontaneous symmetry-breaking. More generally, other kinds of spontaneous symmetry-breaking can occur in the network, depending on its symmetries. For example, in the case of two identical inhibitory populations, two different symmetries may be broken: the symmetry between neurons in a given population, and that between the two populations. In the latter case, the two populations would behave differently from each other, while keeping their corresponding neurons homogeneous. This phenomenon is also characterized by strongly positive intra-population correlations and strongly negative inter-population correlations (result not shown), reinforcing the idea of a general relationship between spontaneous symmetry-breaking and anti-correlations. In Fasoli et al. (2016) we described possible extensions of our formalism to spatially extended networks with more complex symmetries, therefore spontaneous symmetry-breaking is likely to affect also the cross-correlation structure of large-scale neural models.

Negative correlations have been observed in resting-state fMRI experiments, for example during cognitive tasks performed by human subjects (Fox et al., 2005), and also in the frontolimbic circuit of awake rats (Liang et al., 2012), but their origin and functional role are still poorly understood. Our findings suggest branching-point bifurcations and spontaneous symmetry-breaking as a potential neurobiological basis of this phenomenon.

### How pharmacological manipulations may affect the correlation structure of the network

One potential application of our formalism is to model the effect on neural correlations of pharmacological application of drugs that act as agonist or antagonists of the major neurotransmitters (Curtis et al., 1971; Krogsgaard-Larsen et al., 1980; Corda et al., 1992; Chen et al., 1992; Cunningham & Jones 2000; Garcia et al., 2010). The effect of these drugs can be effectively modeled by varying the synaptic strengths in the model; in other words their effect on network dynamics and correlations can be studied through a bifurcation analysis in terms of the parameters *J*_*αβ*_. Our results suggest that pharmacological manipulations that alter synaptic weights of small networks will change their dynamics profoundly. For example whenever for a set of synaptic weights the network does not satisfy the conditions (14), (15), (16), the corresponding bifurcations become forbidden for any pair of stimuli (*I*_*E*_,*I*_*I*_). It is therefore natural to speculate that our formalism may add to theoretical models of the effect of drugs on neural dynamics (Foster et al., 2008).

### Future directions

We studied the cross-correlation structure of multi-population networks near local bifurcations of codimension one. Furthermore, our theory can be easily extended to the analysis of local bifurcations of larger codimension.

Another possible extension of our theory is the study of correlations in sparse networks. In Fasoli et al. (2015) the authors showed that, when the number of connections per neuron does not diverge for *N* →*∞*, asynchronous states in general do not occur in the thermodynamic limit for weak stimuli (compare with van Vreeswijk & Sompolinsky (1998), where the authors considered the case of sparse networks with infinite connections per neuron). Therefore, sufficiently sparse networks cannot rely on their size for generating asynchronous states, but uncorrelated activity can still be generated through strong stimuli or with special combinations of the network’s parameters. Moreover, in Fasoli et al. (2016) we showed that in sparse networks the branching-point bifurcations are more likely to occur, resulting in a considerable increase of the complexity of the bifurcation diagrams.

It is also possible to study correlations in small neural circuits with random synaptic weights, extending the results obtained in Ginzburg & Sompolinsky (1994) and Renart et al. (2010) for large random networks of binary neurons. Their correlation structure can be calculated straightforwardly from the fundamental matrix Φ, by applying the formalism developed in Fasoli et al. (2015) (see Eqs. (4.3) and (4.6) therein). The bifurcation structure of networks with heterogeneous weights has been studied only in the limit of large systems (Hermann & Touboul 2012), and is still unexplored in the case of small neural circuits.

The effect of temporally correlated afferent currents on neural activity has been studied extensively in integrate-and-fire neural network models, see e.g. Brunel & Sergi (1998), Sakai et al. (1999), Moreno et al. (2002), and Renart et al. (2003). We observe that, by following the techniques described in Hennequin et al. (2016), also our finite-size firing-rate model can be easily extended to include temporally correlated noise sources. Due to the non-linear interplay between spatial and temporal correlations, we expect to observe strong quantitative deviations from Eqs. (12) and (13), especially at the bifurcation points of the network. However, in our model these deviations do not affect qualitatively the properties of critical slowing down. In other words, in our model the explosion of the variance of the stochastic fluctuations, and the formation of arbitrarily strong cross- and auto-correlations, are expected to occur regardless of the temporal correlations of the noise sources. Moreover, by extending our perturbative approach to the second order, we would be able to quantify rigorously how temporal correlations affect the skewness of the stochastic fluctuations of the membrane potentials, which is expected to be strongly non-zero at the bifurcation points of the network (Scheffer et al., 2009).

The dependence of network correlations on the neuron’s firing rates has been investigated extensively in recent years (De La Rocha et al., 2007; Ecker et al., 2014; Goris et al., 2014). In particular, for pairs of unconnected cortical neurons receiving correlated inputs *in vitro*, and for model integrate-and-fire neurons, in De La Rocha et al. (2007) the authors reported that correlations increase with the geometric mean of the firing rates. However, in our model we generally observed a non-monotonic dependence of the correlation on the firing rates, that depended on which dynamical state the network was in. In particular, we found non-monotonicity when the stimulus made the network switch between Andronov-Hopf and branching-point bifurcations, or when, given a strong correlation between the Brownian motions, the network was close to a saddle-node bifurcation (see the bottom panels and the top-right panel of Fig. 6, respectively). This result shows that the relation between firing rates and correlations are expected to be more complicated in neuronal networks than in pairs of non-interacting neurons. A consequence of the possibly non-monotonic and dynamical state-dependent relationship between rates and correlations is that the mean firing rates and correlations can act as separate information channels for the encoding of the strength of the external stimuli. A rigorous analysis of the encoding capability of our model may be performed by calculating analytically the Cramér-Rao bound (Abbott & Dayan 1999). In particular, it would be interesting to evaluate how the mean firing rates and the correlation structure of the network contribute to the analytical expression of the Cramér-Rao bound, and to determine the differences in the encoding capability of the network between synchronous and asynchronous regimes. In future work, this approach will allow us to determine systematically whether correlations are detrimental or helpful for the encoding of sensory information in firing-rate network models.

### Online resource 1

#### Theory of correlations for multi-population networks

In this supplemental text we extend our analysis of correlations to networks composed of an arbitrary number of neural populations. In particular, we prove that asynchronous states are elicited by strong stimuli, while synchronous states occur at the local bifurcations of the network, regardless of the number of neural populations. Moreover, we introduce a mathematical formalism for calculating the fundamental matrix Φ of the network, from which the correlation structure can be derived from Eq. (6). For the sake of clarity, we also implemented this formalism with Python in the Online Resource 2.

### Online resource 2

#### Python script 1

In this script we implemented the formalism, described in the Online Resource 1, for the calculation of the fundamental matrix Φ of multi-population neural networks.

### Online resource 3

#### Python script 2

This script performs a comparison between the numerical simulations (described in Section 2.3) and the analytical formulas of the variance and cross-correlation (Eqs. (11)–(13)), in the specific case of the two-population network considered in the main text.

## Electronic supplementary material

Below is the link to the electronic supplementary material.

(PDF 421 KB)

(PY 7.28 KB)

(PY 24.3 KB)

## Footnotes

1 More precisely, the torus bifurcation is a local bifurcation of the Poincaré map of a limit cycle of the network (Kuznetsov 1998). For this reason the torus bifurcation corresponds to a change of stability of the fixed points of the Poincaré map, and not to a change of stability of the equilibrium points of Eq. (1). Therefore, the torus bifurcation cannot be studied through the eigenvalues of the Jacobian matrix of the network.
