# Supplementary material for: Transitions between asynchronous and synchronous states: a theory of correlations in small neural circuits
Source: J Comput Neurosci. 2017 Nov 10;44(1):25–43. doi: 10.1007/s10827-017-0667-3 (PMC5770155; doi:10.1007/s10827-017-0667-3)
Supplement: Supplementary file 1 — (PDF 421 KB) [file 10827_2017_667_MOESM1_ESM.pdf]

Structure of the Supplementary Materials

The Supplementary Materials are organized as follows. First, in Sec. (S1) we introduce the multi-population model we studied. Then, in Sec. (S2) we derive the formula of the corresponding Jacobian matrix $\mathcal{J}$, whose eigenvalues and eigenvectors are calculated in Sec. (S3). In Sec. (S4) we derive the fundamental matrix $\Phi(t) = e^{\mathcal{J}t}$ of the system, while in Sec. (S5) we show the constraints that must be satisfied by the covariance matrix of the noise $\Sigma^{\mathscr{B}}$ in order to be a genuine covariance matrix. Then in Sec. (S6) we use all these results to calculate the cross-correlation structure of the network. In particular, in SubSec. (S6.1) we use the formula of the correlation to prove that the network undergoes a weak-correlation regime with small fluctuations (asynchronous states) when its size is increased to infinity or when the neurons are stimulated by strong inputs. On the other side, in SubSec. (S6.2) we prove that when the network approaches a local bifurcation point, the neural activity undergoes a strong-correlation regime with large fluctuations (critical slowing down). In particular, we discuss only the local bifurcations of codimension one, namely saddle-node, Andronov-Hopf and branching-point bifurcations.

S1 The Multi-Population Model

In this subsection we extend the two-population model of SubSec. (2.1) (see the main text) to the multi-population case. We define N_α to be the size of population α, where $\sum_{\alpha=0}^{\mathfrak{P}-1} N_\alpha = N$ and $\mathfrak{P}$ represents the number of populations in the network. We rearrange the neurons so that the structural connectivity of the network can be written as follows:

$$J = \begin{bmatrix} \mathfrak{J}_{00} & \mathfrak{J}_{01} & \cdots & \mathfrak{J}_{0,\mathfrak{P}-1} \\ \mathfrak{J}_{10} & \mathfrak{J}_{11} & \cdots & \mathfrak{J}_{1,\mathfrak{P}-1} \\ \vdots & \vdots & \ddots & \vdots \\ \mathfrak{J}_{\mathfrak{P}-1,0} & \mathfrak{J}_{\mathfrak{P}-1,1} & \cdots & \mathfrak{J}_{\mathfrak{P}-1,\mathfrak{P}-1} \end{bmatrix}, \qquad \mathfrak{J}_{\alpha\beta} = \begin{cases} J_{\alpha\alpha}\left(\mathbb{I}_{N_\alpha} - \mathrm{Id}_{N_\alpha}\right), & \text{for } \alpha = \beta \\ \\ J_{\alpha\beta}\mathbb{I}_{N_\alpha,N_\beta}, & \text{for } \alpha \neq \beta \end{cases} \tag{S1}$$

for $\alpha, \beta = 0, \ldots, \mathfrak{P}-1$. The real numbers $J_{\alpha\beta}$ are free parameters that describe the strength of the synaptic connections from the population β to the population α. We have $J_{\alpha\beta} \geq 0 \ \forall \alpha$ if the population β is excitatory, and $J_{\alpha\beta} \leq 0 \ \forall \alpha$ if it is inhibitory. The external input currents are organized into $\mathfrak{P}$ vectors $\boldsymbol{I}_\alpha = I_\alpha(t)\,\mathbf{1}_{N_\alpha}$, one for each population, and again the same subdivision between populations is performed for the parameters M, τ, $\nu^{\max}$, Λ, V^T as in the two-population case.

From now on we use the notation $[\mathscr{M}_{\alpha\beta}]_{\forall(\alpha,\beta)}$ to represent a block matrix containing $\mathfrak{P}^2$ blocks $\mathscr{M}_{\alpha\beta}$ for $\alpha, \beta = 0, \ldots, \mathfrak{P}-1$. Thus, for example the synaptic connectivity matrix (see Eq. (S1)) can be written as $J = [\mathfrak{J}_{\alpha\beta}]_{\forall(\alpha,\beta)}$. We use the same notation also for $\mathfrak{P} \times \mathfrak{P}$ matrices. Thus, for example the reduced Jacobian matrix defined later in Eq. (S7) can be written as $\mathcal{J}^{\mathcal{R}} = \left[\mathcal{J}^{\mathcal{R}}_{\alpha\beta}\right]_{\forall(\alpha,\beta)}$.

To conclude the description of the multi-population model, the covariance structure of the white noise $\sigma_i^{\mathscr{B}} \frac{d\mathscr{B}_i(t)}{dt}$ is given by the matrix $\Sigma^{\mathscr{B}} = \left[\varSigma^{\mathscr{B}}_{\alpha\beta}\right]_{\forall(\alpha,\beta)}$, where $\varSigma^{\mathscr{B}}_{\alpha\beta}$ is defined as in Eq. (4) of the main text.

S2 Jacobian Matrix

From Eq. (S1) we get that the full Jacobian matrix of the network is $\mathcal{J} = [\mathscr{J}_{\alpha\beta}]_{\forall(\alpha,\beta)}$, where:

$$\mathscr{J}_{\alpha\beta} = \begin{cases} -\frac{1}{\tau_\alpha}\mathrm{Id}_{N_\alpha} + \frac{J_{\alpha\alpha}}{M_\alpha}\mathscr{A}'_\alpha\left(\mu_\alpha\right)\left(\mathbb{I}_{N_\alpha} - \mathrm{Id}_{N_\alpha}\right), & \text{for } \alpha = \beta \\ \frac{J_{\alpha\beta}}{M_\alpha}\mathscr{A}'_\beta\left(\mu_\beta\right)\mathbb{I}_{N_\alpha,N_\beta}, & \text{for } \alpha \neq \beta. \end{cases} \tag{S2}$$

In Eq. (S2), μ_α are the stationary membrane potentials obtained form Eq. (1) of the main text when the network has constant input I_α and the noise is not present ($\sigma_i^{\mathscr{B}} = 0\ \forall i$). In other words, μ_α are the solutions of the following system of non-linear algebraic equations:

$$-\frac{1}{\tau_\alpha}\mu_\alpha + \frac{N_\alpha - 1}{M_\alpha}J_{\alpha\alpha}\mathscr{A}_\alpha\left(\mu_\alpha\right) + \sum_{\substack{\beta=0\\ \beta\neq\alpha}}^{\mathfrak{P}-1}\frac{N_\beta}{M_\alpha}J_{\alpha\beta}\mathscr{A}_\beta\left(\mu_\beta\right) + I_\alpha = 0, \quad \alpha = 0, \dots, \mathfrak{P} - 1. \tag{S3}$$

Eq. (S3) must be solved numerically, or by the asymptotic perturbative expansion that we introduced in the Supplementary Materials of [1].

S3 Eigenvalues and Eigenvectors

For every $\alpha = 0, \dots, \mathfrak{P} - 1$, it is trivial to prove that the Jacobian matrix (S2) has $N_\alpha - 1$ eigenvalues of the form:

$$\lambda_\alpha^{\mathcal{P}} = -\left[\frac{1}{\tau_\alpha} + \frac{J_{\alpha\alpha}}{M_\alpha}\mathscr{A}'_\alpha\left(\mu_\alpha\right)\right], \tag{S4}$$

with corresponding eigenvectors:

$$\boldsymbol{v}_{\alpha;i}^{\mathcal{P}} = \begin{bmatrix} 0 \\ \vdots \\ 0 \\ 1 \\ \vdots \\ 1 \\ 1 - N_\alpha \\ 1 \\ \vdots \\ 1 \\ 0 \\ \vdots \\ 0 \end{bmatrix}, \quad i = 0, \dots, N_\alpha - 2. \tag{S5}$$

The term $1 - N_\alpha$ is the $(n_{\alpha-1} + i)$th entry of the vector $\boldsymbol{v}_{\alpha;i}^{\mathcal{P}}$, where:

$$n_{\alpha-1} \stackrel{\text{def}}{=} \sum_{p=0}^{\alpha-1} N_p \tag{S6}$$

with $n_{-1} \stackrel{\text{def}}{=} 0$. In Eq. (S5), the $(n_{\alpha-1}+j)$th entries of $\boldsymbol{v}^{\mathcal{P}}_{\alpha;i}$ (with $j=0,\ldots,N_\alpha-1$ and $j\neq i$) are equal to 1, while all the remaining entries are equal to 0.

Now we prove that the remaining $\mathfrak{P}$ eigenvalues of $\mathcal{J}$, that we call $\lambda^{\mathcal{R}}_\alpha$, are those of the $\mathfrak{P}\times\mathfrak{P}$ matrix $\mathcal{J}^{\mathcal{R}} = \left[\mathcal{J}^{\mathcal{R}}_{\alpha\beta}\right]_{\forall(\alpha,\beta)}$ (the "reduced" Jacobian matrix of the network), where:

$$\mathcal{J}^{\mathcal{R}}_{\alpha\beta} = \begin{cases} -\frac{1}{\tau_\alpha} + \frac{N_\alpha - 1}{M_\alpha} J_{\alpha\alpha} \mathscr{A}'_\alpha(\mu_\alpha), & \text{for } \alpha=\beta \\ \frac{N_\beta}{M_\alpha} J_{\alpha\beta}\mathscr{A}'_\beta(\mu_\beta), & \text{for } \alpha\neq\beta \end{cases} \tag{S7}$$

and the corresponding eigenvectors $\boldsymbol{v}^{\mathcal{R}}_\alpha$ are:

$$\boldsymbol{v}^{\mathcal{R}}_\alpha = \begin{bmatrix} \left[\widehat{\boldsymbol{v}}^{\mathcal{R}}_\alpha\right]_0 \\ \vdots \\ \left[\widehat{\boldsymbol{v}}^{\mathcal{R}}_\alpha\right]_0 \\ \vdots \\ \left[\widehat{\boldsymbol{v}}^{\mathcal{R}}_\alpha\right]_{\mathfrak{P}-1} \\ \vdots \\ \left[\widehat{\boldsymbol{v}}^{\mathcal{R}}_\alpha\right]_{\mathfrak{P}-1} \end{bmatrix}. \tag{S8}$$

In Eq. (S8) the entry $\left[\widehat{\boldsymbol{v}}^{\mathcal{R}}_\alpha\right]_\beta$ (namely the βth entry of the vector $\widehat{\boldsymbol{v}}^{\mathcal{R}}_\alpha$) is repeated N_β times, and $\widehat{\boldsymbol{v}}^{\mathcal{R}}_\alpha = \left[\left[\widehat{\boldsymbol{v}}^{\mathcal{R}}_\alpha\right]_0, \left[\widehat{\boldsymbol{v}}^{\mathcal{R}}_\alpha\right]_1, \cdots, \left[\widehat{\boldsymbol{v}}^{\mathcal{R}}_\alpha\right]_{\mathfrak{P}-1}\right]^T$ is the eigenvector of $\mathcal{J}^{\mathcal{R}}$ corresponding to the eigenvalue $\lambda^{\mathcal{R}}_\alpha$.

Proof. By developing the product $\mathcal{J}\boldsymbol{v}^{\mathcal{R}}_\alpha$ the equation $\mathcal{J}\boldsymbol{v}^{\mathcal{R}}_\alpha = \lambda^{\mathcal{R}}_\alpha \boldsymbol{v}^{\mathcal{R}}_\alpha$ can be rewritten as follows:

$$\begin{cases} \sum_{p=0}^{N_0-1} \mathcal{J}_{kp}\left[\boldsymbol{v}^{\mathcal{R}}_\alpha\right]_p + \sum_{p=N_0}^{N_0+N_1-1} \mathcal{J}_{kp}\left[\boldsymbol{v}^{\mathcal{R}}_\alpha\right]_p + \ldots = \lambda^{\mathcal{R}}_\alpha \left[\boldsymbol{v}^{\mathcal{R}}_\alpha\right]_k, & k=0,\ldots,N_0-1 \\ \vdots \\ \sum_{p=0}^{N_0-1} \mathcal{J}_{kp}\left[\boldsymbol{v}^{\mathcal{R}}_\alpha\right]_p + \sum_{p=N_0}^{N_0+N_1-1} \mathcal{J}_{kp}\left[\boldsymbol{v}^{\mathcal{R}}_\alpha\right]_p + \ldots = \lambda^{\mathcal{R}}_\alpha \left[\boldsymbol{v}^{\mathcal{R}}_\alpha\right]_k, & k=N_0+\ldots+N_{\mathfrak{P}-2},\ldots,N-1. \end{cases} \tag{S9}$$

Therefore, if we define:

$$\mathfrak{S}_{\alpha\beta} \stackrel{\text{def}}{=} \sum_{k=n_{\beta-1}}^{n_\beta - 1} \left[\boldsymbol{v}^{\mathcal{R}}_\alpha\right]_k \tag{S10}$$

for $\beta = 0,\ldots,\mathfrak{P}-1$, and if we replace the terms $\mathcal{J}_{kp}$ with the corresponding values given by Eq. (S2), the system (S9) can be rewritten as follows:

$$\sum_{\beta=0}^{\mathfrak{P}-1} \frac{J_{\gamma\beta}}{M_\gamma} \mathscr{A}'_\beta (\mu_\beta)\, \mathfrak{S}_{\alpha\beta} = \left(\frac{1}{\tau_\gamma} + \frac{J_{\gamma\gamma}}{M_\gamma} \mathscr{A}'_\gamma (\mu_\gamma) + \lambda_\alpha^{\mathcal{R}}\right) \left[\boldsymbol{v}_\alpha^{\mathcal{R}}\right]_{k_\gamma}, \qquad k_\gamma = n_{\gamma-1}, ..., n_\gamma - 1 \tag{S11}$$

for $\gamma = 0, \ldots, \mathfrak{P} - 1$. If $\frac{1}{\tau_\gamma} + \frac{J_{\gamma\gamma}}{M_\gamma} \mathscr{A}'_\gamma (\mu_\gamma) + \lambda_\alpha^{\mathcal{R}} \neq 0$, Eq. (S11) implies that $\left[\boldsymbol{v}_\alpha^{\mathcal{R}}\right]_{k_\gamma}$ does not depend on the index k_γ for γ fixed, therefore from Eq. (S10) we get:

$$\mathfrak{S}_{\alpha\beta} = N_\beta \left[\boldsymbol{v}_\alpha^{\mathcal{R}}\right]_{k_\beta}.$$

Thus Eq. (S11) can be rewritten as follows:

$$\sum_{\beta=0}^{\mathfrak{P}-1} \frac{J_{\gamma\beta}}{M_\gamma} \mathscr{A}'_\beta (\mu_\beta)\, N_\beta \left[\boldsymbol{v}_\alpha^{\mathcal{R}}\right]_{k_\beta} = \left(\frac{1}{\tau_\gamma} + \frac{J_{\gamma\gamma}}{M_\gamma} \mathscr{A}'_\gamma (\mu_\gamma) + \lambda_\alpha^{\mathcal{R}}\right) \left[\boldsymbol{v}_\alpha^{\mathcal{R}}\right]_{k_\gamma}. \tag{S12}$$

This is a homogeneous system in the unknowns $\left[\boldsymbol{v}_\alpha^{\mathcal{R}}\right]_{k_\beta}$, with coefficient matrix $\mathcal{J}^{\mathcal{R}} - \lambda_\alpha^{\mathcal{R}} \mathrm{Id}_{\mathfrak{P}}$, where $\mathcal{J}^{\mathcal{R}}$ is given by Eq. (S7). Therefore the system has a non-trivial solution if and only if $\det\left(\mathcal{J}^{\mathcal{R}} - \lambda_\alpha^{\mathcal{R}} \mathrm{Id}_{\mathfrak{P}}\right) = 0$, which defines the characteristic polynomial of the reduced Jacobian matrix. The eigenvalues $\lambda_\alpha^{\mathcal{R}}$ are the roots of this polynomial, with corresponding eigenvectors $\widehat{\boldsymbol{v}}_\alpha^{\mathcal{R}} \overset{\mathrm{def}}{=} \left[\left[\boldsymbol{v}_\alpha^{\mathcal{R}}\right]_{k_0}, \left[\boldsymbol{v}_\alpha^{\mathcal{R}}\right]_{k_1}, \cdots, \left[\boldsymbol{v}_\alpha^{\mathcal{R}}\right]_{k_{\mathfrak{P}-1}}\right]^T$. ■

Note that the rank of the matrix $\mathcal{J}^{\mathcal{R}} - \lambda_\alpha^{\mathcal{R}} \mathrm{Id}_{\mathfrak{P}}$ determines the number of free components $\mathfrak{f}$ of $\boldsymbol{v}_\alpha^{\mathcal{R}}$, through the relation $\mathfrak{f} = \mathfrak{P} - \mathrm{rank}\left(\mathcal{J}^{\mathcal{R}} - \lambda_\alpha^{\mathcal{R}} \mathrm{Id}_{\mathfrak{P}}\right)$ (rank-nullity theorem), with $0 < \mathrm{rank}\left(\mathcal{J}^{\mathcal{R}} - \lambda_\alpha^{\mathcal{R}} \mathrm{Id}_{\mathfrak{P}}\right) < \mathfrak{P}$.

Example for $\mathfrak{P} = 2$

For simplicity, in the case of one excitatory and one inhibitory population discussed in the main text, we call the eigenvalues $\lambda_{0,1}^{\mathcal{P}}$ as $\lambda_{E,I}$, and the corresponding eigenvectors $\boldsymbol{v}_{0,1}^{\mathcal{P}}$ as $\boldsymbol{v}_{E,I}$. Then, according to Eq. (S4), we get:

$$\lambda_E = -\left[\frac{1}{\tau_E} + \frac{J_{EE}}{M_E} \mathscr{A}'_E (\mu_E)\right], \quad \lambda_I = -\left[\frac{1}{\tau_I} + \frac{J_{II}}{M_I} \mathscr{A}'_I (\mu_I)\right], \tag{S13}$$

with multiplicity $N_E - 1$ and $N_I - 1$ respectively, while from Eq. (S5) we obtain:

$$
\boldsymbol{v}_{E;0}=\begin{bmatrix}1-N_E\\1\\\vdots\\1\\1\\0\\0\\\vdots\\0\\0\end{bmatrix},\ \boldsymbol{v}_{E;1}=\begin{bmatrix}1\\1-N_E\\\vdots\\1\\1\\0\\0\\\vdots\\0\\0\end{bmatrix},\ldots,\boldsymbol{v}_{E;N_E-2}=\begin{bmatrix}1\\1\\\vdots\\1-N_E\\1\\0\\0\\\vdots\\0\\0\end{bmatrix},
$$

$$
\boldsymbol{v}_{I;0}=\begin{bmatrix}0\\0\\\vdots\\0\\0\\1-N_I\\1\\\vdots\\1\\1\end{bmatrix},\quad \boldsymbol{v}_{I;1}=\begin{bmatrix}0\\0\\\vdots\\0\\0\\1\\1-N_I\\\vdots\\1\\1\end{bmatrix},\ldots,\quad \boldsymbol{v}_{I;N_I-2}=\begin{bmatrix}0\\0\\\vdots\\0\\0\\1\\1\\\vdots\\1-N_I\\1\end{bmatrix}. \tag{S14}
$$

The remaining eigenvalues of $\mathcal{J}$ are those of the following reduced Jacobian matrix:

$$
\mathcal{J}^{\mathcal{R}}=\begin{bmatrix}-\frac{1}{\tau_E}+\frac{N_E-1}{M_E}J_{EE}\mathscr{A}_E'(\mu_E) & \frac{N_I}{M_E}J_{EI}\mathscr{A}_I'(\mu_I)\\ \frac{N_E}{M_I}J_{IE}\mathscr{A}_E'(\mu_E) & -\frac{1}{\tau_I}+\frac{N_I-1}{M_I}J_{II}\mathscr{A}_I'(\mu_I)\end{bmatrix},
$$

namely:

$$
\lambda_{0,1}^{\mathcal{R}}=\frac{\mathcal{Y}+\mathcal{Z}\pm\sqrt{(\mathcal{Y}-\mathcal{Z})^2+4\mathcal{X}}}{2}, \tag{S15}
$$

where:

$$
\mathcal{X}=\frac{N_EN_I}{M_EM_I}J_{EI}J_{IE}\mathscr{A}_E'(\mu_E)\mathscr{A}_I'(\mu_I),\quad \mathcal{Y}=-\frac{1}{\tau_E}+\frac{N_E-1}{M_E}J_{EE}\mathscr{A}_E'(\mu_E),\quad \mathcal{Z}=-\frac{1}{\tau_I}+\frac{N_I-1}{M_I}J_{II}\mathscr{A}_I'(\mu_I). \tag{S16}
$$

We observe that rank $\left(\mathcal{J}^{\mathcal{R}}-\lambda_\alpha^{\mathcal{R}}\mathrm{Id}_{\mathfrak{P}}\right)=1$, therefore the eigenvectors of $\mathcal{J}^{\mathcal{R}}$ corresponding to the eigenvalues $\lambda_{0,1}^{\mathcal{R}}$ are:

$$
\widehat{\boldsymbol{v}}_0^{\mathcal{R}}=x\begin{bmatrix}1\\K_0\end{bmatrix},\qquad \widehat{\boldsymbol{v}}_1^{\mathcal{R}}=y\begin{bmatrix}1\\K_1\end{bmatrix}, \tag{S17}
$$

where:

$$K_\alpha \stackrel{\text{def}}{=} \frac{M_E\left(\lambda_\alpha^{\mathcal{R}}+\frac{1}{\tau_E}\right)-(N_E-1)\,J_{EE}\mathscr{A}_E'(\mu_E)}{N_I J_{EI}\mathscr{A}_I'(\mu_I)} = \frac{N_E J_{IE}\mathscr{A}_E'(\mu_E)}{M_I\left(\lambda_\alpha^{\mathcal{R}}+\frac{1}{\tau_I}\right)-(N_I-1)\,J_{II}\mathscr{A}_I'(\mu_I)} \tag{S18}$$

(the last equality is a consequence of $\det\left(\mathcal{J}^{\mathcal{R}}-\lambda_\alpha^{\mathcal{R}}\mathrm{Id}_{\mathfrak{P}}\right)=0$). Moreover, x, y are two parameters that represent the free components of $\widehat{\boldsymbol{v}}_{0,1}^{\mathcal{R}}$ (one for each eigenvector, according to the relation $\mathfrak{f}=\mathfrak{P}-\mathrm{rank}\left(\mathcal{J}^{\mathcal{R}}-\lambda_\alpha^{\mathcal{R}}\mathrm{Id}_{\mathfrak{P}}\right)=1$). Therefore the corresponding eigenvectors of $\mathcal{J}$ are:

$$\boldsymbol{v}_0^{\mathcal{R}} = x\begin{bmatrix}1\\1\\ \vdots\\1\\K_0\\K_0\\ \vdots\\K_0\end{bmatrix}, \qquad \boldsymbol{v}_1^{\mathcal{R}} = y\begin{bmatrix}1\\1\\ \vdots\\1\\K_1\\K_1\\ \vdots\\K_1\end{bmatrix}, \tag{S19}$$

where the first N_E entries of $\boldsymbol{v}_{0,1}^{\mathcal{R}}$ in Eq. (S19) are equal to 1 and thus $K_{0,1}$ are the remaining N_I entries.

S4 Fundamental Matrix

When $\mathcal{J}$ is diagonalizable, its powers can be written as $\mathcal{J}^n=PD^nP^{-1}$, where:

$$\begin{aligned} D &= \mathrm{diag}\left(\lambda_0^{\mathcal{P}},\ldots,\lambda_{\mathfrak{P}-1}^{\mathcal{P}},\lambda_0^{\mathcal{R}},\ldots,\lambda_{\mathfrak{P}-1}^{\mathcal{R}}\right)\\ D^n &= \mathrm{diag}\left(\left(\lambda_0^{\mathcal{P}}\right)^n,\ldots,\left(\lambda_{\mathfrak{P}-1}^{\mathcal{P}}\right)^n,\left(\lambda_0^{\mathcal{R}}\right)^n,\ldots,\left(\lambda_{\mathfrak{P}-1}^{\mathcal{R}}\right)^n\right), \end{aligned}$$

while P is an $N\times N$ matrix whose columns are composed of the eigenvectors of $\mathcal{J}$ calculated in Sec. (S3). If we introduce the matrices:

$$\mathit{\Psi}_{\alpha\beta;n} = \begin{cases} \Xi_{\alpha;n}\mathrm{Id}_{N_\alpha}+\Gamma_{\alpha;n}\left(\mathbb{I}_{N_\alpha}-\mathrm{Id}_{N_\alpha}\right), & \text{for } \alpha=\beta\\ \Omega_{\alpha\beta;n}\mathbb{I}_{N_\alpha,N_\beta}, & \text{for } \alpha\neq\beta \end{cases} \tag{S20}$$

such that $\mathcal{J}^n=\left[\mathit{\Psi}_{\alpha\beta;n}\right]_{\forall(\alpha,\beta)}$, and we replace Eq. (S20) and the matrix P (expressed in terms of the eigenvectors of $\mathcal{J}$) into the formula $\mathcal{J}^nP=PD^n$, after some algebra we get the following set of equations:

$$\begin{cases} N_\alpha\left[\widehat{\boldsymbol{v}}_\gamma^{\mathcal{R}}\right]_\alpha\Gamma_{\alpha;n}+\displaystyle\sum_{\substack{\beta=0\\ \beta\neq\alpha}}^{\mathfrak{P}-1}N_\beta\left[\widehat{\boldsymbol{v}}_\gamma^{\mathcal{R}}\right]_\beta\Omega_{\alpha\beta;n}=\left[\widehat{\boldsymbol{v}}_\gamma^{\mathcal{R}}\right]_\alpha\left(\left(\lambda_\gamma^{\mathcal{R}}\right)^n-\left(\lambda_\alpha^{\mathcal{P}}\right)^n\right)\\ \Xi_{\alpha;n}-\Gamma_{\alpha;n}=\left(\lambda_\alpha^{\mathcal{P}}\right)^n \end{cases} \tag{S21}$$

for $\gamma = 0, \ldots, \mathfrak{P}-1$. Now if we define:

$$\Omega_{\alpha\alpha;n} \stackrel{\text{def}}{=} \Gamma_{\alpha;n} + \frac{1}{N_\alpha}\left(\lambda_\alpha^{\mathcal{P}}\right)^n, \tag{S22}$$

the first equation of the system (S21) becomes:

$$\sum_{\beta=0}^{\mathfrak{P}-1} N_\beta \left[\widehat{\boldsymbol{v}}_\gamma^{\mathcal{R}}\right]_\beta \Omega_{\alpha\beta;n} = \left[\widehat{\boldsymbol{v}}_\gamma^{\mathcal{R}}\right]_\alpha \left(\lambda_\gamma^{\mathcal{R}}\right)^n, \quad \gamma = 0, \ldots, \mathfrak{P}-1.$$

In matrix form the system reads $\mathfrak{A}\mathfrak{x}_{\alpha;n} = \mathfrak{b}_{\alpha;n}$, where:

$$\begin{gathered}\mathfrak{A} = \left(P^{\mathcal{R}}\right)^T \operatorname{diag}\left(N_0, N_1, \cdots, N_{\mathfrak{P}-1}\right) \\ \mathfrak{x}_{\alpha;n} = \begin{bmatrix} \Omega_{\alpha 0;n} \\ \Omega_{\alpha 1;n} \\ \vdots \\ \Omega_{\alpha,\mathfrak{P}-1;n} \end{bmatrix}, \quad \mathfrak{b}_{\alpha;n} = \begin{bmatrix} \left[\widehat{\boldsymbol{v}}_0^{\mathcal{R}}\right]_\alpha \left(\lambda_0^{\mathcal{R}}\right)^n \\ \left[\widehat{\boldsymbol{v}}_1^{\mathcal{R}}\right]_\alpha \left(\lambda_1^{\mathcal{R}}\right)^n \\ \vdots \\ \left[\widehat{\boldsymbol{v}}_{\mathfrak{P}-1}^{\mathcal{R}}\right]_\alpha \left(\lambda_{\mathfrak{P}-1}^{\mathcal{R}}\right)^n \end{bmatrix}, \quad \alpha = 0, \ldots, \mathfrak{P}-1.\end{gathered} \tag{S23}$$

In Eq. (S23), $P^{\mathcal{R}}$ is a $\mathfrak{P} \times \mathfrak{P}$ matrix whose columns are composed of the eigenvectors of $\mathcal{J}^{\mathcal{R}}$, namely:

$$P^{\mathcal{R}} = \begin{bmatrix} \left[\widehat{\boldsymbol{v}}_0^{\mathcal{R}}\right]_0 & \left[\widehat{\boldsymbol{v}}_1^{\mathcal{R}}\right]_0 & \cdots & \left[\widehat{\boldsymbol{v}}_{\mathfrak{P}-1}^{\mathcal{R}}\right]_0 \\ \left[\widehat{\boldsymbol{v}}_0^{\mathcal{R}}\right]_1 & \left[\widehat{\boldsymbol{v}}_1^{\mathcal{R}}\right]_1 & \cdots & \left[\widehat{\boldsymbol{v}}_{\mathfrak{P}-1}^{\mathcal{R}}\right]_1 \\ \vdots & \vdots & \ddots & \vdots \\ \left[\widehat{\boldsymbol{v}}_0^{\mathcal{R}}\right]_{\mathfrak{P}-1} & \left[\widehat{\boldsymbol{v}}_1^{\mathcal{R}}\right]_{\mathfrak{P}-1} & \cdots & \left[\widehat{\boldsymbol{v}}_{\mathfrak{P}-1}^{\mathcal{R}}\right]_{\mathfrak{P}-1} \end{bmatrix}.$$

Therefore by inverting the system $\mathfrak{A}\mathfrak{x}_{\alpha;n} = \mathfrak{b}_{\alpha;n}$ we obtain:

$$\Omega_{\alpha\beta;n} = \sum_{\gamma=0}^{\mathfrak{P}-1} \mathfrak{C}_{\alpha\beta}^{(\gamma)} \left(\lambda_\gamma^{\mathcal{R}}\right)^n,$$

where:

$$\mathfrak{C}_{\alpha\beta}^{(\gamma)} = \left[\mathfrak{A}^{-1}\right]_{\beta\gamma} \left[\widehat{\boldsymbol{v}}_\gamma^{\mathcal{R}}\right]_\alpha = \frac{1}{N_\beta}\left[\left(P^{\mathcal{R}}\right)^{-T}\right]_{\beta\gamma} \left[\widehat{\boldsymbol{v}}_\gamma^{\mathcal{R}}\right]_\alpha, \quad \left(P^{\mathcal{R}}\right)^{-T} \stackrel{\text{def}}{=} \left[\left(P^{\mathcal{R}}\right)^T\right]^{-1}. \tag{S24}$$

We observe that the matrix $\mathfrak{C}^{(\gamma)} \stackrel{\text{def}}{=} \left[\mathfrak{C}^{(\gamma)}_{\alpha\beta}\right]_{\forall(\alpha,\beta)}$ can be written as an outer product of vectors, namely:

$$\mathfrak{C}^{(\gamma)} = \begin{bmatrix} \left[\widehat{\boldsymbol{v}}^{\mathcal{R}}_{\gamma}\right]_0 \\ \left[\widehat{\boldsymbol{v}}^{\mathcal{R}}_{\gamma}\right]_1 \\ \vdots \\ \left[\widehat{\boldsymbol{v}}^{\mathcal{R}}_{\gamma}\right]_{\mathfrak{P}-1} \end{bmatrix} \left[\left[\mathfrak{A}^{-1}\right]_{0\gamma}, \left[\mathfrak{A}^{-1}\right]_{1\gamma}, \cdots, \left[\mathfrak{A}^{-1}\right]_{\mathfrak{P}-1,\gamma}\right], \tag{S25}$$

therefore it has rank one. This result will prove very important in demonstrating the formation of critical slowing down near the saddle-node bifurcations (see SubSec. (S6.2.1)).

Moreover, from the second equation of (S21) and from the definition (S22), we get:

$$\Xi_{\alpha;n} = \left(\lambda^{\mathcal{P}}_{\alpha}\right)^n + \Gamma_{\alpha;n} = \left(1 - \frac{1}{N_\alpha}\right)\left(\lambda^{\mathcal{P}}_{\alpha}\right)^n + \Omega_{\alpha\alpha;n}.$$

Therefore we can rewrite Eq. (S20) as follows:

$$\varPsi_{\alpha\beta;n} = \begin{cases} \left(\sum_{\gamma=0}^{\mathfrak{P}-1} \mathfrak{C}^{(\gamma)}_{\alpha\alpha}\left(\lambda^{\mathcal{R}}_{\gamma}\right)^n\right)\mathbb{I}_{N_\alpha} + \left(-\frac{1}{N_\alpha}\mathbb{I}_{N_\alpha} + \mathrm{Id}_{N_\alpha}\right)\left(\lambda^{\mathcal{P}}_{\alpha}\right)^n, & \text{for } \alpha = \beta \\ \left(\sum_{\gamma=0}^{\mathfrak{P}-1} \mathfrak{C}^{(\gamma)}_{\alpha\beta}\left(\lambda^{\mathcal{R}}_{\gamma}\right)^n\right)\mathbb{I}_{N_\alpha,N_\beta}, & \text{for } \alpha \neq \beta. \end{cases} \tag{S26}$$

To conclude, from the Taylor expansion $e^{\mathcal{J}t} = \sum_{n=0}^{\infty} \frac{t^n}{n!}\mathcal{J}^n$ and Eq. (S26) we get $\Phi(t) = \left[\varPhi_{\alpha\beta}(t)\right]_{\forall(\alpha,\beta)}$, where:

$$\varPhi_{\alpha\beta}(t) = \begin{cases} \left(\sum_{\gamma=0}^{\mathfrak{P}-1} \mathfrak{C}^{(\gamma)}_{\alpha\alpha} e^{\lambda^{\mathcal{R}}_{\gamma} t}\right)\mathbb{I}_{N_\alpha} + \left(-\frac{1}{N_\alpha}\mathbb{I}_{N_\alpha} + \mathrm{Id}_{N_\alpha}\right) e^{\lambda^{\mathcal{P}}_{\alpha} t}, & \text{for } \alpha = \beta \\ \left(\sum_{\gamma=0}^{\mathfrak{P}-1} \mathfrak{C}^{(\gamma)}_{\alpha\beta} e^{\lambda^{\mathcal{R}}_{\gamma} t}\right)\mathbb{I}_{N_\alpha,N_\beta}, & \text{for } \alpha \neq \beta. \end{cases} \tag{S27}$$

For the sake of clarity, we implemented this method in the Online Resource 2 for an arbitrary number of populations. Furthermore, below we show the explicit calculation of the fundamental matrix in the case $\mathfrak{P} = 2$.

Example for $\mathfrak{P} = 2$

In the case of two neural populations, from Eq. (S17) we get $P^{\mathcal{R}} = \begin{bmatrix} 1 & 1 \\ K_0 & K_1 \end{bmatrix}$ and therefore $\left(P^{\mathcal{R}}\right)^{-T} = \frac{1}{K_1 - K_0}\begin{bmatrix} K_1 & -K_0 \\ -1 & 1 \end{bmatrix}$. Thus, according to Eqs. (S24) and (S27), the blocks of the fundamental matrix $\Phi(t) = \begin{bmatrix} \varPhi_{EE}(t) & \varPhi_{EI}(t) \\ \varPhi_{IE}(t) & \varPhi_{II}(t) \end{bmatrix}$ are given by the following formulas:

$$\Phi_{EE}(t)=\frac{K_1e^{\lambda_0^{\mathcal{R}}t}-K_0e^{\lambda_1^{\mathcal{R}}t}}{N_E(K_1-K_0)}\mathbb{I}_{N_E}+\left(-\frac{1}{N_E}\mathbb{I}_{N_E}+\mathrm{Id}_{N_E}\right)e^{\lambda_E t},\qquad \Phi_{EI}(t)=\frac{e^{\lambda_1^{\mathcal{R}}t}-e^{\lambda_0^{\mathcal{R}}t}}{N_I(K_1-K_0)}\mathbb{I}_{N_E,N_I},$$

$$\Phi_{II}(t)=\frac{K_1e^{\lambda_1^{\mathcal{R}}t}-K_0e^{\lambda_0^{\mathcal{R}}t}}{N_I(K_1-K_0)}\mathbb{I}_{N_I}+\left(-\frac{1}{N_I}\mathbb{I}_{N_I}+\mathrm{Id}_{N_I}\right)e^{\lambda_I t},\qquad \Phi_{IE}(t)=\frac{K_0K_1\left(e^{\lambda_0^{\mathcal{R}}t}-e^{\lambda_1^{\mathcal{R}}t}\right)}{N_E(K_1-K_0)}\mathbb{I}_{N_I,N_E}. \tag{S28}$$

S5 Constraints for the Covariance Matrix of the Noise

In order to be a genuine covariance matrix, $\Sigma^{\mathscr{B}}$ must be positive-semidefinite. Being symmetric, $\Sigma^{\mathscr{B}}$ is positive-semidefinite if and only if its eigenvalues are non-negative. Since $\Sigma^{\mathscr{B}}$ has the same block structure of the Jacobian matrix, we can easily calculate its eigenvalues by adapting the results of Sec. (S3). For example, in the case $\mathfrak{P}=2$ we get that the matrix $\Sigma^{\mathscr{B}}$ has to satisfy the following set of inequalities in order to be positive-semidefinite:

$$\begin{cases}\left(\sigma_E^{\mathscr{B}}\right)^2\left[1+(N_E-1)\,C_{EE}^{\mathscr{B}}\right]+\left(\sigma_I^{\mathscr{B}}\right)^2\left[1+(N_I-1)\,C_{II}^{\mathscr{B}}\right]\geq 0\\ \left[1+(N_E-1)\,C_{EE}^{\mathscr{B}}\right]\left[1+(N_I-1)\,C_{II}^{\mathscr{B}}\right]\geq N_EN_I\left(C_{EI}^{\mathscr{B}}\right)^2.\end{cases}$$

The importance of this constraint is highlighted by the following relation:

$$\sum_{i,j=0}^{N-1}x_ix_j\mathrm{Cov}\left(\sigma_i^{\mathscr{B}}\frac{d\mathscr{B}_i(t)}{dt},\sigma_j^{\mathscr{B}}\frac{d\mathscr{B}_j(t)}{dt}\right)=\mathrm{Var}\left(\sum_{i=0}^{N-1}x_i\sigma_i^{\mathscr{B}}\frac{d\mathscr{B}_i(t)}{dt}\right).$$

Indeed, from this equality we see that if $\Sigma^{\mathscr{B}}$ were not positive-semidefinite, the linear combination $\sum_{i=0}^{N-1}x_i\sigma_i^{\mathscr{B}}\frac{d\mathscr{B}_i(t)}{dt}$ would have negative variance for some coefficients x_i.

S6 Correlation Structure

Now we have all the ingredients for studying the phenomena that affects the correlation structure of the network. In particular, in this section we prove that, depending on the parameters of the network, the neural activity spans from strong anti-correlation (i.e. $\mathrm{Corr}(V_i(t),V_j(t))\to\frac{1}{1-N_\gamma}$ for some inhibitory population γ) to strongly positive correlation ($\mathrm{Corr}(V_i(t),V_j(t))\to 1$), passing through arbitrarily weak correlation ($\mathrm{Corr}(V_i(t),V_j(t))\to 0$). We consider the weak-correlation regime in SubSec. (S6.1), while we study the strong-correlation regime in SubSec. (S6.2) in relation to the local bifurcations of the network. For both the regimes, we analyze the cross-correlation for an arbitrary number of populations, extending the results of the case $\mathfrak{P}=2$ that we developed in the main text (see Eqs. (12) + (13)) without the need of explicit formulas. Indeed, we show that it is possible to evaluate the qualitative behavior of the covariance matrix of the membrane potentials Σ^V even if the explicit expressions of the coefficients $\mathfrak{C}_{\alpha\beta}^{(\gamma)}$ in Eq. (S27) are not known.

S6.1 Asynchronous Regime

According to the mean-field theory developed by McKean, Tanaka and Sznitman [2–9], the cross-correlation of the multi-population network in the thermodynamic limit ($N_\alpha \to \infty \ \forall \alpha$) tends to zero for every pair of neurons if the Brownian motions are independent (i.e. $C^{\mathscr{B}}_{\alpha\beta} = 0 \ \forall \alpha, \beta$). This result was proven in [10] for an arbitrary number of neural populations with infinite size.

However, having an infinite size is not the only way a network can experience low levels of correlation. As explained in [11], for strongly depolarizing or strongly hyperpolarizing stimuli the activation function saturates to $\nu^{\max}$ or 0 respectively (see Eq. (2) of the main text), therefore $\mathscr{A}'(V) \to 0$. Given a network with an arbitrary number of populations of arbitrary size, if the stimulus is strong enough for all the populations, the Jacobian matrix of the network (see Eq. (S2)) becomes diagonal. In other words, the neurons become *effectively* disconnected. For this reason the correlation structure of the membrane potentials is determined only by the Brownian motions, namely $\mathrm{Corr}(V_i(t), V_j(t)) \to C^{\mathscr{B}}_{\alpha\beta}$ for every pair of neurons i, j in the populations α, β, respectively. Therefore, given arbitrary (i.e. non-specifically tuned) network parameters, the necessary requirement to generate weak correlation between neurons is the absence of noise correlations, namely $C^{\mathscr{B}}_{\alpha\beta} = 0 \ \forall \alpha, \beta$. This mechanism is different from that described in [10], since it occurs for $|I_\alpha| \to \infty$ rather than $N_\alpha \to \infty$, and it allows the network to create decorrelated activity even if its size is small.

S6.2 Synchronous Regime

Interestingly, the network is able to show also high values of correlation, either positive or negative. This phenomenon occurs at the (local) bifurcation points of the network, and therefore for special combinations of the network's parameters. As we explained in the main text, we consider only the codimension one bifurcations of the network, namely saddle-node, Andronov-Hopf and branching-point bifurcations. Since we want to prove that critical slowing down is due to the synaptic connections and that it occurs regardless the presence of correlation between the Brownian motions, it suffices to study the correlation structure near the bifurcations points in the case $C^{\mathscr{B}}_{\alpha\beta} = 0 \ \forall \alpha, \beta$. In this case, Eq. (6) of the main text reduces to:

$$\mathrm{Cov}(V_i(t), V_j(t)) = \sum_{k=0}^{N-1} \left(\sigma^{\mathscr{B}}_k\right)^2 \int_0^t \Phi_{ik}(t-s)\,\Phi_{jk}(t-s)\,ds. \tag{S29}$$

The relation between the bifurcations and the correlation structure of the network is described in the next subsections by means of Eq. (S29). The analysis can be extended to the case of correlated noise by using Eq. (6) of the main text, but this does not add anything new to the results (compare with the case $\mathfrak{P} = 2$ considered in the main text).

S6.2.1 Saddle-Node Bifurcations

The multi-population neural network undergoes saddle-node bifurcations when one of the eigenvalues of the reduced Jacobian matrix tends to zero [1]. Therefore, if $\lambda^{\mathcal{R}}_\gamma \to 0^-$ for a given γ, and all the other eigenvalues have negative real part, then for $t \gg \frac{1}{|\lambda^{\mathcal{R}}_\gamma|}$ Eq. (S27) gives:

$$\mathit{\Phi}_{\alpha\beta}(t) \approx \mathfrak{C}^{(\gamma)}_{\alpha\beta} e^{-|\lambda^{\mathcal{R}}_\gamma| t} \mathbb{I}_{N_\alpha, N_\beta} \quad \forall \alpha, \beta.$$

According to Eq. (S29), this implies:

$$\mathrm{Cov}\left(V_i\left(t\right),V_j\left(t\right)\right)\approx\frac{1-e^{-2\left|\lambda_\gamma^{\mathcal{R}}\right|t}}{2\left|\lambda_\gamma^{\mathcal{R}}\right|}\sum_{\beta=0}^{\mathfrak{P}-1}N_\beta\left(\sigma_\beta^{\mathscr{B}}\right)^2\mathfrak{C}_{\mathfrak{p}(i),\beta}^{(\gamma)}\mathfrak{C}_{\mathfrak{p}(j),\beta}^{(\gamma)},\ \forall i,j, \tag{S30}$$

where $\mathfrak{p}\left(k\right)$ represents the neural population the kth neuron belongs to. Now, since $\mathrm{Var}\left(V_i\left(t\right)\right)=\mathrm{Cov}\left(V_i\left(t\right),V_i\left(t\right)\right)\approx\frac{1}{2\left|\lambda_\gamma^{\mathcal{R}}\right|}\sum_{\beta=0}^{\mathfrak{P}-1}N_\beta\left(\sigma_\beta^{\mathscr{B}}\mathfrak{C}_{\mathfrak{p}(i),\beta}^{(\gamma)}\right)^2$ for $t\gg\frac{1}{\left|\lambda_\gamma^{\mathcal{R}}\right|}$, the amplitude of the fluctuations diverges for $\lambda_\gamma^{\mathcal{R}}\to0^-$. Moreover, if $\mathfrak{p}\left(i\right)=\mathfrak{p}\left(j\right)$, Eq. (S30) gives $\mathrm{Cov}\left(V_i\left(t\right),V_j\left(t\right)\right)\approx\mathrm{Var}\left(V_i\left(t\right)\right)=\mathrm{Var}\left(V_j\left(t\right)\right)$, therefore according to Pearson's formula $\mathrm{Corr}\left(V_i\left(t\right),V_j\left(t\right)\right)\approx1$. On the other side, if $\mathfrak{p}\left(i\right)\neq\mathfrak{p}\left(j\right)$, by replacing Eq. (S30) within the condition $\mathrm{Cov}^2\left(V_i\left(t\right),V_j\left(t\right)\right)=\mathrm{Var}\left(V_i\left(t\right)\right)\mathrm{Var}\left(V_j\left(t\right)\right)$, after some algebra we get:

$$\sum_{\substack{\beta_0,\beta_1=0\\\beta_0<\beta_1}}^{\mathfrak{P}-1}N_{\beta_0}N_{\beta_1}\left(\sigma_{\beta_0}^{\mathscr{B}}\sigma_{\beta_1}^{\mathscr{B}}\right)^2\left(\mathfrak{C}_{\mathfrak{p}(i),\beta_0}^{(\gamma)}\mathfrak{C}_{\mathfrak{p}(j),\beta_1}^{(\gamma)}-\mathfrak{C}_{\mathfrak{p}(i),\beta_1}^{(\gamma)}\mathfrak{C}_{\mathfrak{p}(j),\beta_0}^{(\gamma)}\right)^2=0.$$

This equality is satisfied if and only if:

$$\mathfrak{C}_{\mathfrak{p}(i),\beta_0}^{(\gamma)}\mathfrak{C}_{\mathfrak{p}(j),\beta_1}^{(\gamma)}-\mathfrak{C}_{\mathfrak{p}(i),\beta_1}^{(\gamma)}\mathfrak{C}_{\mathfrak{p}(j),\beta_0}^{(\gamma)}=\det\left(\left[\begin{array}{cc}\mathfrak{C}_{\mathfrak{p}(i),\beta_0}^{(\gamma)}&\mathfrak{C}_{\mathfrak{p}(i),\beta_1}^{(\gamma)}\\\mathfrak{C}_{\mathfrak{p}(j),\beta_0}^{(\gamma)}&\mathfrak{C}_{\mathfrak{p}(j),\beta_1}^{(\gamma)}\end{array}\right]\right)=0\quad\forall\beta_0,\beta_1,$$

namely if and only if the matrix $\mathfrak{C}^{(\gamma)}$ has rank 1, which is indeed the case (see Sec. (S4)). This proves the emergence of strong correlations also between the neurons of different populations when the network is close to a saddle-node bifurcation.

S6.2.2 Andronov-Hopf Bifurcations

The multi-population neural network undergoes an Andronov-Hopf bifurcation whenever the reduced Jacobian matrix has two complex-conjugate purely imaginary eigenvalues [1]. If the real part of a pair of complex-conjugate eigenvalues $\lambda_{\gamma,\delta}^{\mathcal{R}}$ tends to zero (i.e. $\Re\left(\lambda_\gamma^{\mathcal{R}}\right)\to0^-$), and if all the other eigenvalues have negative real part, then for $t\gg\frac{1}{\left|\Re\left(\lambda_\gamma^{\mathcal{R}}\right)\right|}$ Eq. (S27) gives:

$$\varPhi_{\alpha\beta}\left(t\right)\approx\left(\mathfrak{C}_{\alpha\beta}^{(\gamma)}e^{\lambda_\gamma^{\mathcal{R}}t}+\overline{\mathfrak{C}}_{\alpha\beta}^{(\gamma)}e^{\overline{\lambda}_\gamma^{\mathcal{R}}t}\right)\mathbb{I}_{N_\alpha,N_\beta}\quad\forall\alpha,\beta,$$

where $\overline{\lambda}_\gamma^{\mathcal{R}}=\lambda_\delta^{\mathcal{R}}$ and the overline represents the complex conjugate operator. According to Eq. (S29), after some algebra we get:

$$\mathrm{Cov}\left(V_i\left(t\right),V_j\left(t\right)\right)\approx\frac{1-e^{-2\left|\Re\left(\lambda_\gamma^{\mathcal{R}}\right)\right|t}}{\left|\Re\left(\lambda_\gamma^{\mathcal{R}}\right)\right|}\sum_{\beta=0}^{\mathfrak{P}-1}N_\beta\left(\sigma_\beta^{\mathscr{B}}\right)^2\Re\left(\mathfrak{C}_{\mathfrak{p}(i),\beta}^{(\gamma)}\overline{\mathfrak{C}}_{\mathfrak{p}(j),\beta}^{(\gamma)}\right),\ \forall i,j \tag{S31}$$

thus, similarly to the case of the saddle-node bifurcations, for $t \gg \frac{1}{\left|\Re\left(\lambda_\gamma^{\mathcal{R}}\right)\right|}$ the amplitude of the fluctuations diverges for $\Re\left(\lambda_\gamma^{\mathcal{R}}\right) \rightarrow 0^-$. Moreover, if $\mathfrak{p}(i) = \mathfrak{p}(j)$, Eq. (S31) gives $\mathrm{Cov}\left(V_i(t), V_j(t)\right) \approx \mathrm{Var}\left(V_i(t)\right) = \mathrm{Var}\left(V_j(t)\right)$, therefore $\mathrm{Corr}\left(V_i(t), V_j(t)\right) \approx 1$. On the other side, if $\mathfrak{p}(i) \neq \mathfrak{p}(j)$, from Eq. (S31) it is possible to prove that the condition $\mathrm{Cov}^2\left(V_i(t), V_j(t)\right) = \mathrm{Var}\left(V_i(t)\right)\mathrm{Var}\left(V_j(t)\right)$ is satisfied if and only if $\Im\left(\mathfrak{C}_{\mathfrak{p}(i),\beta}^{(\gamma)}\right) = \Im\left(\mathfrak{C}_{\mathfrak{p}(j),\beta}^{(\gamma)}\right) = 0 \; \forall\beta$. However, these imaginary parts cannot be equal to zero at an Andronov-Hopf bifurcation, the latter being defined by a pair of purely imaginary eigenvalues. Therefore, contrary to the saddle-node bifurcations, the cross-correlation between neurons in different populations does not tend to one at an Andronov-Hopf bifurcation. This is also confirmed by the top-right panel of Fig. (4) in the main text, in the special case $\mathfrak{P} = 2$.

S6.2.3 Branching-Point Bifurcations

Branching-point bifurcations occur whenever $\lambda_\gamma^{\mathcal{P}} \rightarrow 0^-$ for a given γ [1]. According to Eq. (S4), only sufficiently strong self-inhibitory synaptic weights $J_{\gamma\gamma}$ give rise to this kind of bifurcation. As we explained in [1], branching points do not occur in the mean-field approximation of the network, therefore the study of correlations near these bifurcations is one of the results of most interest of our article.

If $\lambda_\gamma^{\mathcal{P}} \rightarrow 0^-$ and all the other eigenvalues have negative real part, for $t \gg \frac{1}{\left|\lambda_\gamma^{\mathcal{P}}\right|}$ Eq. (S27) gives:

$$\varPhi_{\gamma\beta}(t) \approx \begin{cases} \left(-\frac{1}{N_\gamma}\mathbb{I}_{N_\gamma} + \mathrm{Id}_{N_\gamma}\right) e^{-\left|\lambda_\gamma^{\mathcal{P}}\right| t}, & \text{for } \gamma = \beta \\ \mathbb{O}_{N_\gamma, N_\beta}, & \text{for } \gamma \neq \beta, \end{cases} \tag{S32}$$

where $\mathbb{O}_{N_\gamma, N_\beta}$ is the $N_\gamma \times N_\beta$ null matrix. According to Eq. (S29), if $\mathfrak{p}(i) = \mathfrak{p}(j) = \gamma$ we get:

$$\mathrm{Cov}\left(V_i(t), V_j(t)\right) \approx \frac{1 - e^{-2\left|\lambda_\gamma^{\mathcal{P}}\right| t}}{2\left|\lambda_\gamma^{\mathcal{P}}\right|}\left(\sigma_\gamma^{\mathscr{B}}\right)^2\left(-\frac{1}{N_\gamma}\right), \text{ for } i \neq j \tag{S33}$$

$$\mathrm{Var}\left(V_i(t)\right) = \mathrm{Var}\left(V_j(t)\right) \approx \frac{1 - e^{-2\left|\lambda_\gamma^{\mathcal{P}}\right| t}}{2\left|\lambda_\gamma^{\mathcal{P}}\right|}\left(\sigma_\gamma^{\mathscr{B}}\right)^2\left(1 - \frac{1}{N_\gamma}\right), \tag{S34}$$

thus for $t \gg \frac{1}{\left|\lambda_\gamma^{\mathcal{P}}\right|}$ the amplitude of the fluctuations diverges for $\lambda_\gamma^{\mathcal{P}} \rightarrow 0^-$, while $\mathrm{Corr}\left(V_i(t), V_j(t)\right) \approx \frac{1}{1-N_\gamma}$. Interestingly, according to [11] this is the lower bound of the correlation between fully-connected neurons in a homogeneous population of size N_γ. Since $\frac{1}{1-N_\gamma} < 0$ for $N_\gamma \geq 2$, the neurons in population γ are maximally anti-correlated at the branching-point bifurcations. Moreover, according to this formula, correlation tends to -1 only for $N_\gamma = 2$ (which is confirmed by the top-right panel of Fig. (4) in the main text, in the special case $\mathfrak{P} = 2$).

To conclude, if $\mathfrak{p}(i) = \mathfrak{p}(j) \neq \gamma$, the matrix $\varPhi_{\mathfrak{p}(i),\mathfrak{p}(i)}(t)$ is not dominated by the term $e^{-\left|\lambda_\gamma^{\mathcal{P}}\right| t}$, therefore we do not observe neither the divergence of the variance nor strong correlations in population $\mathfrak{p}(i)$. This prevents also the formation of strong inter-population correlations between γ and $\mathfrak{p}(i)$. In the case $\mathfrak{P} = 2$ this phenomenon occurs for the excitatory neurons since $\gamma = I$ and $\mathfrak{p}(i) = E$, and indeed this result is confirmed by the top panels of Fig. (4) in the main text.

References

[1] Fasoli D, Cattani A, Panzeri S. The complexity of dynamics in small neural circuits. PLoS Comput Biol. 2016;**12**(8):e1004992.

[2] McKean H. A class of Markov processes associated with nonlinear parabolic equations. PNAS. 1966;**56**(6):1907–1911.

[3] McKean H. Propagation of chaos for a class of non-linear parabolic equations. In: Stochastic differential equations (lecture series in differential equations, session 7, Catholic Univ., 1967). Air Force Office Sci. Res., Arlington, Va.; 1967. p. 41–57.

[4] Tanaka H. Probabilistic treatment of the Boltzmann equation of Maxwellian molecules. Probab Theory Rel. 1978;**46**:67–105.

[5] Tanaka H. Central limit theorem for a simple diffusion model of interacting particles. Hiroshima Math J. 1981;**11**(2):415–423.

[6] Tanaka H. Some probabilistic problems in the spatially homogeneous Boltzmann equation. In: Kallianpur G, editor. Theory and application of random fields. vol. **49** of Lect. Notes Contr. Inf. Sci. Springer Berlin Heidelberg; 1983. p. 258–267.

[7] Sznitman A. Nonlinear reflecting diffusion process, and the propagation of chaos and fluctuations associated. J Funct Anal. 1984;**56**:311–336.

[8] Sznitman A. A propagation of chaos result for Burgers' equation. Probab Theory Rel. 1986;**71**:581–613.

[9] Sznitman A. Topics in propagation of chaos. In: Hennequin PL, editor. Ecole d'eté de probabilités de Saint-Flour XIX – 1989. vol. **1464** of Lect. Notes Math. Springer Berlin Heidelberg; 1991. p. 165–251.

[10] Touboul J, Hermann G, Faugeras O. Noise-induced behaviors in neural mean field dynamics. SIAM J Appl Dyn Syst. 2012;**11**(1):49–81.

[11] Fasoli D, Faugeras O, Panzeri S. A formalism for evaluating analytically the cross-correlation structure of a firing-rate network model. J Math Neurosci. 2015;**5**:6.
